# Genome-Wide Identification and Analysis of Auxin Response Factor Transcription Factor Gene Family in *Populus euphratica*

**DOI:** 10.3390/plants14081248

**Published:** 2025-04-19

**Authors:** Yunzhu Shi, Zixuan Mu, Xiangyu Meng, Xiang Li, Lingxuan Zou, Xuli Zhu, Wenhao Bo

**Affiliations:** 1 State Key Laboratory of Tree Genetics and Breeding, National Engineering Research Center of Tree Breeding and Ecological Restoration, Key Laboratory of Genetics and Breeding in Forest Trees and Ornamental Plants, Ministry of Education, College of Biological Sciences and Biotechnology, Beijing Forestry University, Beijing 100083, China; yunzhushi202@bjfu.edu.cn (Y.S.); zxuanmu@163.com (Z.M.); mengxiangyu1302@163.com (X.M.); 18228938307@163.com (X.L.); zoulingxuan@foxmail.com (L.Z.); 2 Center for Computational Biology, College of Biological Sciences and Technology, Beijing Forestry University, Beijing 100083, China

**Keywords:** ARF gene family, auxin response factor, *Populus euphratica*, genomics

## Abstract

Auxin response factor (ARF) is a plant-specific transcription factor that responds to changes in auxin levels, regulating various biological processes in plants such as flower development, senescence, lateral root formation, stress response, and secondary metabolite accumulation. In this study, we identified the ARF gene family in *Populus euphratica* Oliv. using bioinformatics analysis, examining their conserved structural domains, gene structure, expression products, and evolutionary relationships. We found that the 34 *PeARF* genes were unevenly distributed on 19 chromosomes of *P. euphratica*. All 56 PeARF proteins were hydrophilic and unstable proteins localized in the nucleus, with secondary structures containing α-helices, extended strands, random coils, and β-turns but lacking transmembrane helices (TM-helices) and signal peptides. Evolutionary analysis divided the PeARF proteins into five subfamilies (A–E), with high conservation observed in the order and number of motifs, domains, gene structure, and other characteristics within each subfamily. Expression pattern analysis revealed that 17 *PeARF* genes were upregulated during cell growth and heterophylly development. This comprehensive analysis provides insights into the molecular mechanisms of ARF genes in *P. euphratica* growth, development, and stress response, serving as a basis for further studies on the auxin signaling pathway in *P. euphratica*.

## 1. Introduction

Auxin response factors (ARFs) are pivotal transcription factors in plant cells, playing a central role in the auxin signal transduction pathway, thereby regulating plant growth and development [1,2,3]. ARF transcription factors directly regulate downstream gene expression by binding to specific DNA sequences known as auxin response elements (AREs), influencing the formation, elongation, differentiation, and various physiological processes of plant organs [4,5]. The expression and activity of ARF transcription factors family members are tightly regulated through mechanisms such as post-transcriptional modification, protein stability, and signal-dependent degradation [6]. This precise regulation ensures that plants can adaptively respond to different developmental stages and environmental conditions. With the advancements in molecular biology techniques, we have a better understanding of the function of ARF transcription factors and their role in plant growth and response to environmental stress [7,8,9,10]. By modulating the expression pattern or activity of ARF transcription factors, it is possible to regulate plant growth and development and enhance plant tolerance to stress [11,12]. This indicates that ARF transcription factors hold significant application potential in agriculture and forestry production.

The ARF gene family’s involvement in leaf development and other processes in herbaceous plants is well established, but its specific function in woody species requires further investigation. As a representative species, 39 members of the ARF gene family have been identified in *Populus trichocarpa* [13]. Yang et al. also identified 20 members of the ARF gene family in a hybrid line between *Populus deltoides* and *Populus euramericana* [14]. These findings suggest that the ARF gene family is widespread in *Populus* and exhibits strong evolutionary conservation.

*Populus euphratica* Oliv. is the only natural tree species that thrives in the desert regions of northwest China [15,16]. Renowned for its resilience to cold, heat, salt, alkali, sand, and drought [17,18,19], this species plays a crucial role in windbreak and sand fixation and maintaining ecological equilibrium [20,21]. *P. euphratica* is a dioecious species primarily pollinated by wind, which contributes to its high genetic diversity across populations. These biological traits make it a valuable resource for studying genetic adaptation and functional differentiation in response to extreme environments. In addition, as a dicotyledonous species, *P. euphratica* shares an evolutionary lineage with the model plant *Arabidopsis thaliana*. This evolutionary relationship offers a useful reference framework for exploring gene families in *P. euphratica* based on insights gained from well-characterized model species. It is the preferred tree species for vegetation restoration and artificial afforestation in arid desert regions. The completion of the genome sequencing of *P. euphratica* in 2013 has provided a comprehensive dataset, enabling research on this species at the whole-genome level and laying a foundation for functional genomics research [22]. While several gene families such as DREB, MYB, and AP2/ERF have been identified and analyzed in *P. euphratica* [23,24,25], the ARF gene family has not yet been studied. Genome-wide identification and analysis of the ARF gene family in *P. euphratica* can enhance our understanding of the molecular mechanisms underlying ARF gene regulation in growth, development, and stress response. Furthermore, it can provide a theoretical reference for a systems biology study of the auxin signaling pathway in *P. euphratica* in the future.

This study aims to analyze and predict the basic characteristics, gene and protein structures, physicochemical properties, and evolutionary relationships of the *P. euphratica* ARF transcription factor gene family at the genome-wide level. This will be achieved through gene structure analysis, protein structure analysis, functional prediction, and evolutionary analysis, based on the genome-wide sequencing data of *P. euphratica*. The findings of this study will serve as a reference for further elucidating the molecular mechanisms by which ARF genes in *P. euphratica* participate in growth, development, and responses to stress. Additionally, it will provide a theoretical basis for a systems biology study of the auxin signaling pathway in *P. euphratica*.

## 2. Materials and Methods

### 2.1. Experimental Material

The datasets used in this study include the HMM profile of ARF transcription factors from the Pfam database (InterPro) and the ARF protein sequences of *A. thaliana* from the TAIR database. Additionally, the *P. euphratica* PopEup1.0 version whole-genome second-generation sequencing data, uploaded by Ma in 2013, were downloaded from the NCBI database [22]. Transcriptome FPKM (fragments per kilobase of exon model per million mapped fragments) data of *P. euphratica* young heteromorphic leaves’ (linear leaves, lanceolate leaves, ovate leaves, and broadly ovate leaves) tissues were also downloaded from the NCBI GEO (Gene Expression Omnibus) database (https://www.ncbi.nlm.nih.gov/geo/query/acc.cgi?acc=GSE120822, accessed on 12 February 2023). These four types of heteromorphic leaves represent sequential developmental stages from young to mature leaf forms, with linear leaves being the youngest and broadly ovate leaves the most mature.

### 2.2. Identification of the ARF Gene Family in P. euphratica

First, the ARF gene family sequences of *A. thaliana* were retrieved from the TAIR database (https://www.arabidopsis.org/, accessed on 12 February 2023). These sequences were then used as queries to perform BLAST 1.3.0 (basic local alignment search tool) searches against the *P. euphratica* genome in the NCBI database (https://www.ncbi.nlm.nih.gov/, accessed on 15 February 2023) to identify potential ARF homologs. To further validate candidate sequences, the HMM profile of ARF-specific domains was downloaded from the Pfam database via InterPro 104.0 (https://www.ebi.ac.uk/interpro/, accessed on 15 February 2023) and used to confirm the presence of conserved ARF domains in the predicted proteins. The resulting genes were named PeARF1 to PeARF34 based on their e-values, sorted in ascending order. Alternatively spliced isoforms corresponding to the same gene locus were distinguished by isoform numbers (Xn), as annotated in the NCBI database.

### 2.3. Prediction of the Physical and Chemical Properties of Proteins

We used the ProtParam online tool, available on the ExPasy website (https://web.expasy.org/protparam/, accessed on 3 April 2023), to calculate the physicochemical properties of PeARF proteins. Specifically, we calculated parameters including molecular weight, theoretical isoelectric point (pI), instability index, aliphatic index, lipophilicity index, and the grand average of hydropathicity (GRAVY). These values provide insight into the stability, hydrophobicity, and potential solubility of the predicted proteins.

### 2.4. Protein Tertiary Structure Prediction and Subcellular Localization Analysis

The SWISS-MODEL online tool (https://swissmodel.expasy.org/, accessed on 5 April 2023) was used for predicting the tertiary structure of ARF proteins. To assess the degree of structural similarity among different PeARF proteins, TM-align was employed to perform pairwise structural alignments and calculate TM-scores, RMSD values (Å), and sequence identity percentages. The TMHMM 1.0.24 online tool (https://dtu.biolib.com/DeepTMHMM, accessed on 5 April 2023) was used for predicting the transmembrane protein structure of ARF proteins. Finally, the WOLF PSORT online tool (https://wolfpsort.hgc.jp/, accessed on 5 April 2023 ) was used for predicting the subcellular localization of *P. euphratica* ARF proteins.

### 2.5. Construction and Analysis of the Evolutionary Tree

The amino acid sequence information of the ArARF transcription factor gene family proteins was downloaded from the *A. thaliana* database (TAIR). The amino acid sequences of the ARF proteins of P. euphratica and A. thaliana were aligned using the ClustalW algorithm in MEGA 5.0. An evolutionary tree was constructed using the neighbor-joining (NJ) method with p-distance as the amino acid substitution model and 1000 bootstrap replications, with bootstrap values displayed at the nodes. Finally, the tree was visualized using the EvolView online tool (https://www.evolgenius.info/evolview/, accessed on 3 April 2023).

### 2.6. Protein Conserved Motif Prediction

The conserved motif of the ARF protein in *P. euphratica* was analyzed using the MEME 5.1.2 online tool (https://meme-suite.org/meme/, accessed on 16 February 2023). Additionally, the NCBI Batch CD-Search tool (https://www.ncbi.nlm.nih.gov/Structure/bwrpsb/bwrpsb.cgi, accessed on 18 May 2023) was used to assess the constitutive relationship between conserved domains and motifs.

### 2.7. Chromosome Localization Analysis

The chromosomal locations of the *P. euphratica* ARF genes were determined by aligning their genomic coordinates to the chromosome-level assembly of *P. euphratica* (PopEup1.0). This alignment was based on scaffold-to-chromosome mapping and SNP-based anchoring information provided by Beijing Forestry University, which was used to assemble scaffolds into chromosome-scale pseudomolecules. The online tool MapGene2 Chrom web v2 (http://mg2c.iask.in/mg2c_v2.0/, accessed on 4 April 2023) was utilized for chromosome localization analysis and visualization.

### 2.8. Analysis of Gene Structure and cis-Acting Elements

TBtools-II (v1.120) software was employed to extract the coding sequence (CDS) information of the ARF gene family from *P. euphratica* genome sequencing and annotation files. Additionally, the nucleotide sequence information of the 2000 bp upstream promoter region of the ARF gene family in *P. euphratica* was obtained. The online tool PlantCARE (https://bioinformatics.psb.ugent.be/webtools/plantcare/html/, accessed on 6 April 2023) and TBtools software were used for visualizing and analyzing the cis-acting regulatory elements in the aforementioned results. Furthermore, the “Visualize Gene Structure” function of TBtools software and the GSDS online website (http://gsds.gao-lab.org) were utilized to analyze the gene structure of ARF in *P. euphratica*, resulting in the determination of exon and intron distributions.

### 2.9. Analysis of Expression Patterns

The transcriptome FPKM data of leaf tissue from various types of young heteromorphic leaves (including linear leaves, lanceolate leaves, ovate leaves, and broadly ovate leaves) were obtained from the NCBI GEO database (accession number: GSE120822). These RNA-seq datasets were derived from the same genotype and variety of *P. euphratica*, collected from a single plant individual, thereby minimizing potential variation related to the dioecious nature of the species. All analyses in this section were conducted using bioinformatics methods. An R script written in R (v4.2.2) was developed to screen and process the FPKM values of the ARF gene in *P. euphratica*. Following logarithm conversion standardization with a base of 2, the R packages pheatmap and ggplot2 were used to draw heat maps. Subsequently, the relationship between the PeARF gene family and the development of heterophylly was analyzed.

## 3. Results

### 3.1. Identification and Physicochemical Properties of the ARF Gene Family in P. euphratica

Through BLAST and Pfam domain analysis, we identified 56 ARF proteins in *P. euphratica* that simultaneously contain both AuxRE and B3 (DBD) domains, corresponding to 34 ARF genes. These proteins and genes were named PeARF1 to PeARF34, respectively (Table S1). Analysis of the amino acid sequences revealed substantial variation in protein length, ranging from 592 amino acids (PeARF32) to 1131 amino acids (PeARF21). The predicted molecular weights of PeARF proteins span from 65,303.76 Da to 126,034.12 Da. The isoelectric point (pI) values varied between 5.35 (PeARF24) and 9.02 (PeARF19), with the majority of proteins being acidic; only seven proteins have pI values above 7.0. The aliphatic index, which reflects the relative volume of aliphatic side chains (Ala, Val, Leu, Ile) and relates to thermostability, ranged from 64.06 to 78.58. The GRAVY (grand average of hydropathicity) values ranged from −0.68 to −0.294, indicating that all PeARF proteins are hydrophilic. The instability index ranged from 43.78 (PeARF19) to 71.85 (PeARF16-X4). As all values exceed the standard threshold of 40, these proteins are predicted to be structurally unstable in vitro.

### 3.2. Protein Tertiary Structure and Subcellular Localization Prediction

**Figure 1:**
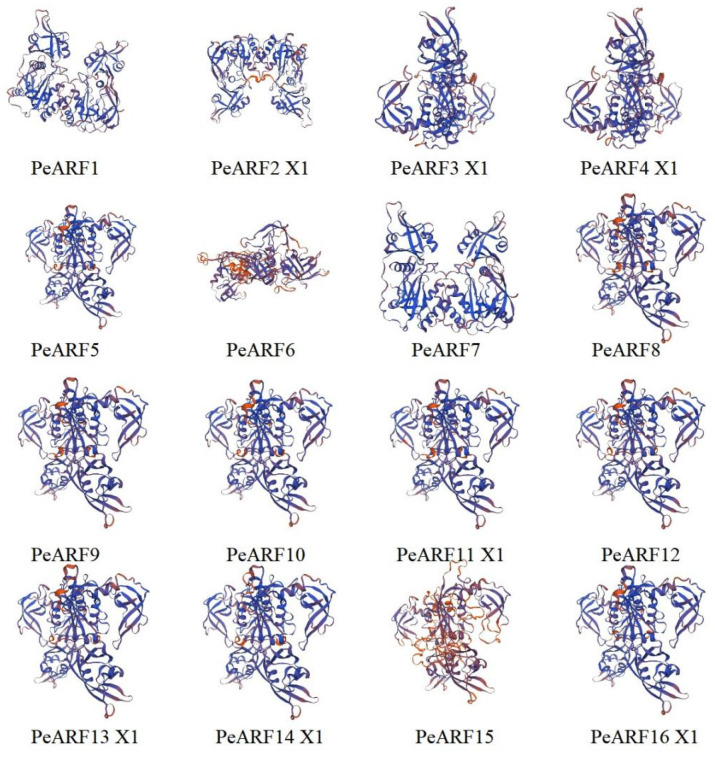
Predicted tertiary structures of ARF family members in *P. euphratica* (partial results; full set is shown in Figure S1). Each panel represents a different ARF protein; in these diagrams, alpha-helices are typically shown as coiled ribbons, beta-sheets as arrows pointing in the direction of the beta-strands, and loops or turns as lines or ropes connecting the secondary structures. Blue coloring represents the protein backbone, and the orange and red elements represent regions of potential functional significance.

According to the predicted secondary structure of PeARF proteins (Table S2), all proteins are composed of four typical structural elements: α-helix, β-turn, extended strand, and random coil. However, their relative proportions differ substantially among family members. Random coil accounts for the highest proportion, ranging from 44.77% to 68.20%, with the lowest proportion in PeARF16 X1 and the highest in PeARF23 X2. The β-turn proportion is the lowest, ranging from 3.08% to 8.53%, with the lowest proportion in PeARF14 X1 and the highest in PeARF16 X3. The tertiary structures of PeARF proteins were further analyzed to assess structural similarity. As shown in Table S3, the TM-scores of pairwise comparisons ranged from 0.65 to 0.95 (mean: 0.80), indicating a generally conserved 3D fold among PeARF members. The RMSD values ranged from 1.00 Å to 3.49 Å (mean: 2.25 Å), while sequence identity spanned 30.01% to 94.98% (mean: 62.90%), reflecting moderate-to-high structural and sequence similarity. Partial results of the predicted tertiary structure of PeARF proteins are shown in Figure 1 (complete results are in Figure S1); combined with the structural similarity analysis in Table S3, these results indicate that PeARF proteins exhibit similar tertiary conformations, primarily composed of random coil regions. Using the TMHMM website to analyze whether members of the PeARF transcription factor gene family have transmembrane structures, we found that all PeARF proteins lack transmembrane helices (TMRs) or signal peptides, indicating that they are all intracellular proteins. Using the WOLF PSORT tool for subcellular localization prediction of PeARF genes (Table S4), the results showed that all PeARF family proteins were located in the cell nucleus, which was consistent with the prediction results of subcellular localization, suggesting that *P. euphratica* ARF proteins play a role in the cell nucleus.

### 3.3. Evolutionary Classification of PeARF and AtARF Gene Families

**Figure 2:**
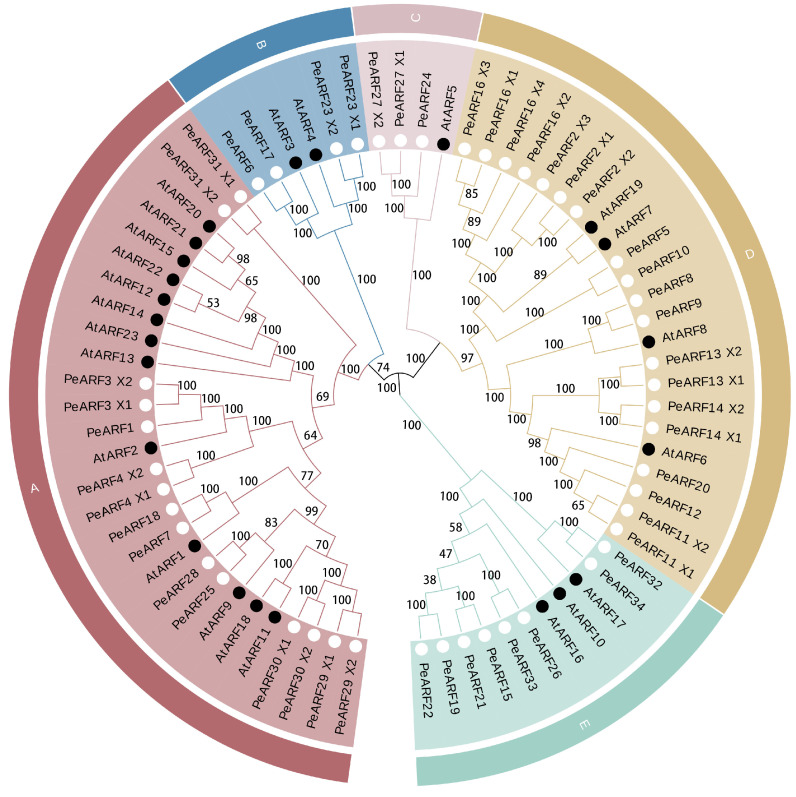
Evolutionary tree of ARF transcription factor proteins in *P. euphratica* and *A. thaliana*. Different colors represent different subgroups within the ARF family. Subgroup A contains 15 PeARF and 13 AtARF members; subgroup B includes 4 PeARF and 2 AtARF members; subgroup C consists of 3 PeARF and 1 AtARF member; subgroup D comprises 19 PeARF and 4 AtARF members; and subgroup E contains 8 PeARF and 3 AtARF members. The branch pattern illustrates the evolutionary relationships among ARF proteins, where closer proximity between two proteins indicates a closer evolutionary relationship. Bootstrap values from 1000 replicates are displayed at each node to indicate the reliability of the clustering. Black dots indicate ARF proteins derived from A. thaliana, while white dots indicate ARF proteins derived from *P. euphratica*.

To investigate the relationship between *P. euphratica* ARF proteins and those of *A. thaliana*, we used the MEGA 5.0 software to construct an evolutionary tree. The clustering analysis results are presented in Figure 2, where the 34 PeARF and 23 AtARF members are categorized into five subfamilies, denoted alphabetically as subfamilies A to E. The closer the evolutionary relationship, the more similar the protein sequence and structure. Subgroup A is the largest, comprising 15 PeARF and 13 AtARF members. Subgroup B includes four PeARF and two AtARF members, while subgroup C is the smallest, consisting of three PeARF members and one AtARF member. Subgroup D comprises 19 PeARF and 4 AtARF members, and subgroup E contains eight PeARF and three AtARF members. Each subfamily contains members from both *P. euphratica* and *A. thaliana*, with the PeARF to AtARF member ratios ranging from 15:13 to 19:4.

### 3.4. Structural Features of Conserved Motifs

**Figure 3:**
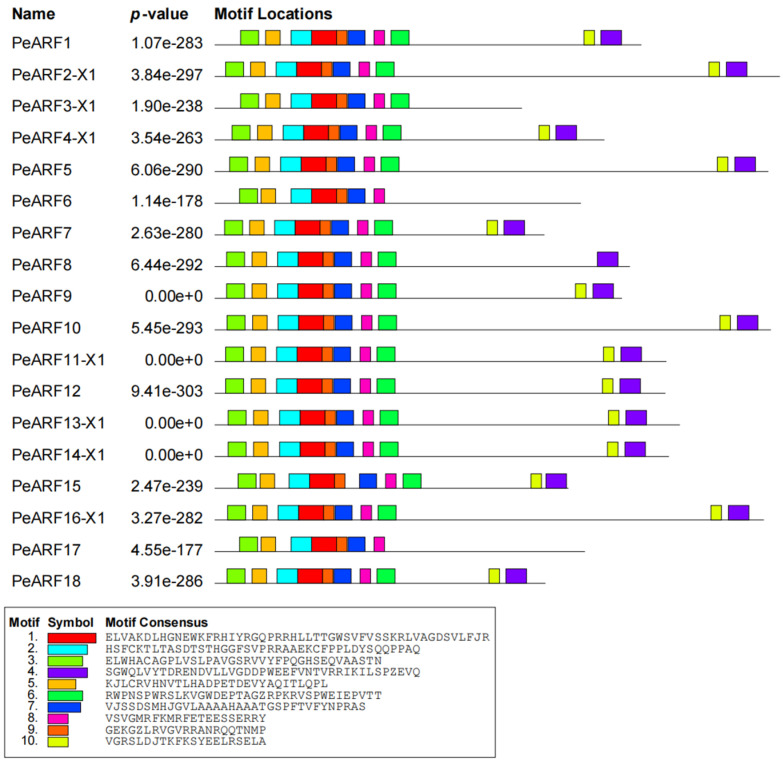
Conserved motifs of *P. euphratica* ARF transcription factor proteins. Different colors represent different motifs (see legend), and the position and length of blocks reflect the specific position and size of each conserved motif in the protein sequence.

Using the MEME online program, we conducted an analysis of the conserved motifs present in PeARF proteins. A partial result is depicted in Figure 3, with complete results available in Figure S2. Our analysis identified 10 conserved motifs, denoted as Motif1 to Motif10, with varying lengths and amino acid compositions. Motif1, Motif2, and Motif3 were highly conserved and widely distributed across nearly all PeARF proteins, suggesting their fundamental functional roles in the ARF family. The partial results of multiple sequence alignment for *P. euphratica* ARF proteins are illustrated in Figure S3. These results reveal that Motif1 and Motif2 collectively form the first B3 (DBD) domain, while Motif5, Motif6, Motif8, and Motif10 amalgamate to construct the second AuxRE sequence-binding domain. Additionally, Motif4 and Motif9 combine to shape the third Aux_IAA binding domain. These findings demonstrate the conserved presence and structural arrangement of motifs and domains within PeARF proteins.

### 3.5. Chromosomal Distribution Patterns of PeARF Genes

**Figure 4:**
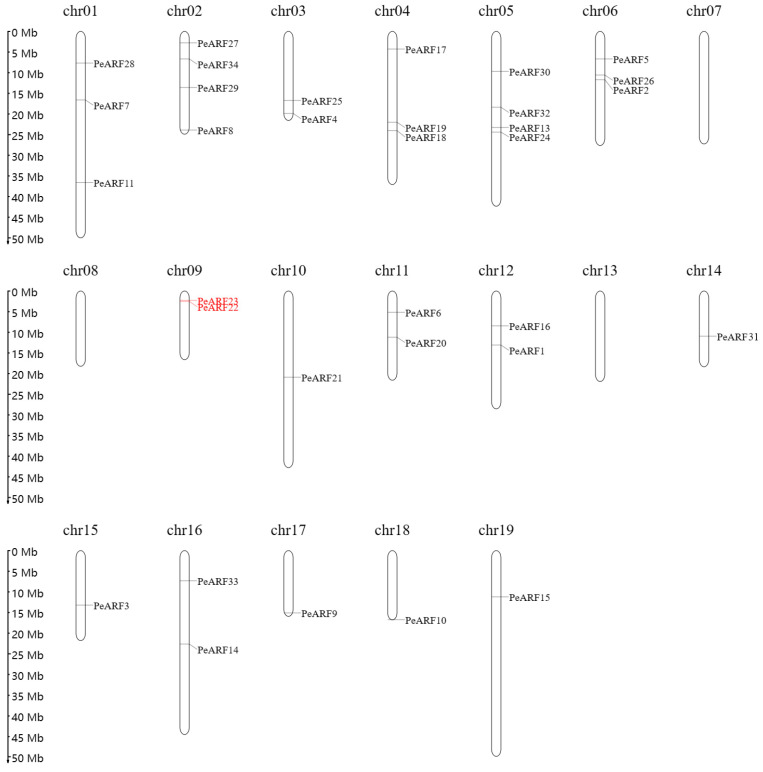
Chromosomal distribution of PeARF genes in *P. euphratica*. The 33 identified PeARF genes are mapped onto 19 chromosomes. Black-labeled genes are inferred to have originated from whole-genome or segmental duplications based on their evolutionary clustering in Figure 2 and their dispersed chromosomal positions. Red-labeled genes (PeARF22 and PeARF23) are physically adjacent on Chr09 and belong to the same evolutionary subgroup, suggesting tandem duplication.

The analysis successfully located 33 genes on 19 chromosomes of *P. euphratica*, except for PeARF12, which could not be assigned to any known scaffold or chromosome. Using MapGene2 Chrom web v2 software, the chromosomal positions of 33 ARF genes in *P. euphratica* were determined. As illustrated in Figure 4, the distribution of *PeARF* gene family members on the chromosomes varied, with chromosomes 2 and 5 having the largest number of *PeARF* genes, totaling four. Chromosomes 7, 8, and 13 had no *PeARF* gene distribution. Notably, PeARF22 and PeARF23 are located adjacent to each other on chromosome 9 and belong to the same evolutionary subclade (Figure 2), supporting their classification as tandem duplicates.

### 3.6. Gene Structure Diversity and Promoter cis-Acting Element Landscape

**Figure 5:**
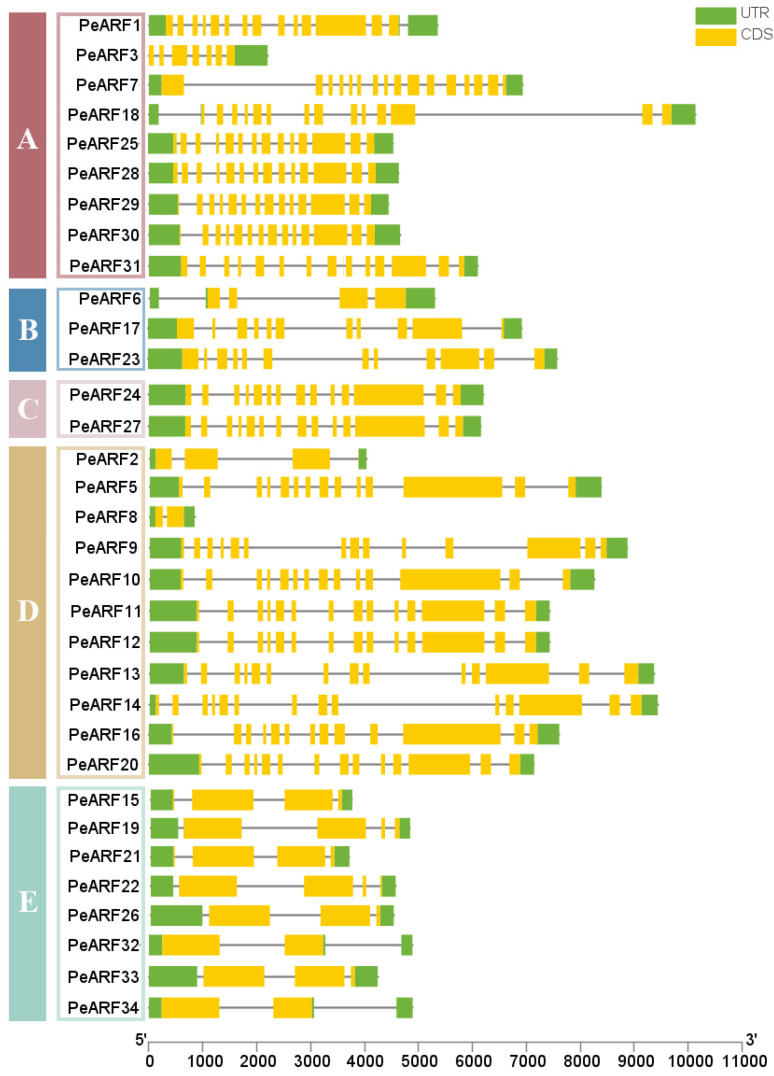
ARF gene family structure of *P. euphratica*, with each horizontal bar representing one gene, the yellow region representing coding sequence, and green region representing untranslated region. The genes are grouped according to their evolutionary subfamilies (**A**–**E**, as defined in Figure 2), shown on the left.

As illustrated in Figure 5, the *PeARF* genes in *P. euphratica* contain 2 to 16 exons and 2 to 15 introns. All 34 *PeARF* genes exhibit intronic structures, with 5′-UTR and 3′-UTR non-coding regions. Among them, *PeARF8* has the shortest exon length, whereas *PeARF10* has the longest. The longest non-coding region is *PeARF11*, while the shortest is *PeARF2*. As shown in Figure 5, members within the same evolutionary subfamily exhibit notable similarities in gene structure, including exon number, exon length, and distribution, indicating a conserved evolutionary pattern. In contrast, genes from different subfamilies display greater structural diversity, suggesting that PeARF genes have undergone subfamily-specific structural divergence during evolution.

**Figure 6:**
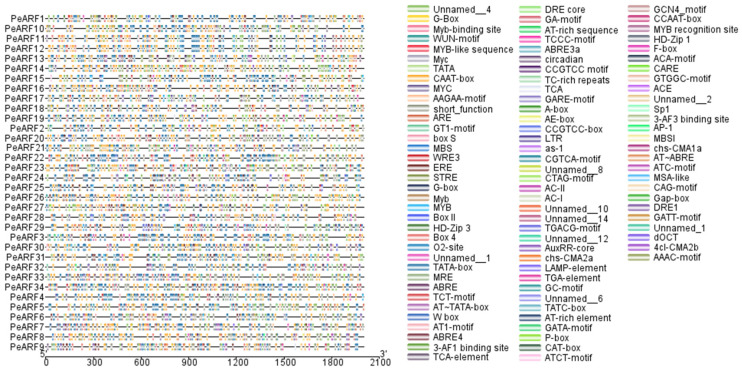
Distribution of cis-acting elements of *P. euphratica* ARF gene family. Different colored squares represent different cis-acting elements, with the legend of cis-acting elements on the right side, where different colors correspond to the names of elements in the legend. Elements labeled as “Unnamed” refer to motifs detected through sequence pattern matching that have not been assigned a specific functional annotation in the PlantCARE database.

The PlantCARE online tool was utilized to analyze the 2000 bp promoter sequence upstream of the *PeARF* gene, with the results depicted in Figure 6. In addition to core promoter elements such as TATA-box and CAAT-box, a wide range of functional cis-acting elements were identified. Among these, hormone-responsive elements, including AuxRR-core, ABRE, and ERE, were the most abundant, with 299 occurrences (5.56%). Light-responsive elements, such as Box 4 and G-box, were observed 326 times (6.06%), while stress-related elements, including STRE and MBS, appeared 290 times (5.39%). A detailed breakdown of the frequency and proportion of hormone-, light-, and stress-responsive cis-acting elements is provided in Supplementary Table S5. These findings suggest that the *PeARF* gene is regulated not only hormones like auxin, abscisic acid, and ethylene but also by light and other environmental conditions, thereby regulating the growth, development, and environmental adaptation processes of *P. euphratica*. The upstream promoter elements of different *PeARF* genes exhibit slight variations.

### 3.7. Expression Dynamics of PeARF Genes During Leaf Morphogenesis

**Figure 7:**
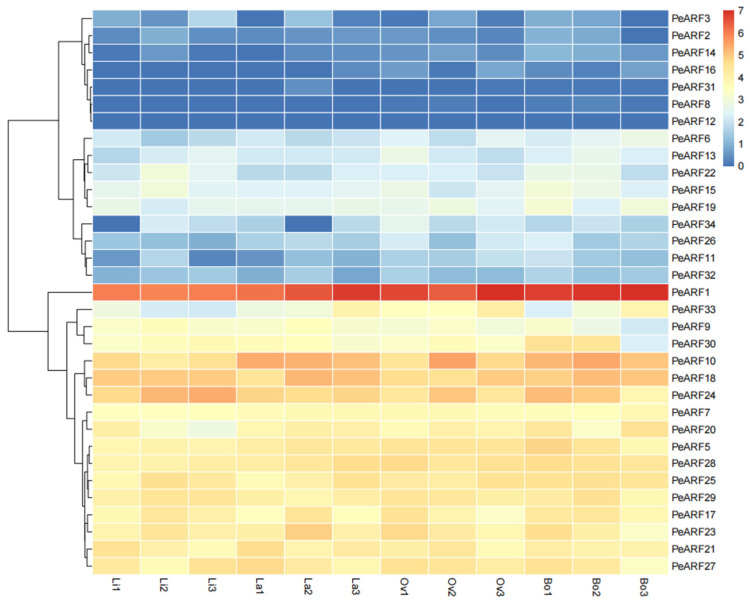
Differential expression of ARF gene in leaves with different morphologies of *P. euphratica*. The expression levels are represented by FPKM values after log2 transformation, with red indicating high expression level, yellow indicating moderate expression level, and blue indicating low expression level. “Li” represents linear leaves, “La” represents lanceolate leaves, “Ov” represents ovate leaves, and “Bo” represents broad ovate leaves.

The expression patterns of *PeARF* genes across different leaf morphologies were visualized as a heatmap (Figure 7), revealing dynamic transcriptional changes during heterophylly development. Among the 34 *ARF* genes of *P. euphratica*, 33 were detected in the transcriptome sequencing of young leaves, with only *PeARF4* showing no expression. A group of genes including *PeARF1*, *PeARF33*, and *PeARF9* exhibited relatively high expression, particularly in later stages of leaf development. In contrast, *PeARF6*, *PeARF13*, and *PeARF22* were expressed at lower levels. Several genes such as *PeARF3*, *PeARF2*, *PeARF14*, *PeARF16*, *PeARF31*, *PeARF8*, and *PeARF12* showed very low or undetectable expression across the leaf types.

## 4. Discussion

In recent years, research on the ARF transcription factor gene family has primarily focused on *Arabidopsis*, tomato, rice, and other plants [26,27,28,29], with relatively little research on the *P. euphratica* ARF transcription factor gene family. In order to better understand the function of *P. euphratica* ARF genes, this study employed bioinformatics methods to identify ARF genes throughout the genome of *P. euphratica*. A total of 34 *P. euphratica* ARF genes and 56 *P. euphratica* ARF proteins were obtained at the genome-wide level, exceeding the numbers in *A. thaliana* and rice. This suggests that new functions have been introduced into the *P. euphratica* ARF family during evolution. The physicochemical properties of the ARF proteins of *P. euphratica* showed minimal differences among *P. euphratica* ARF proteins, all of which were identified as unstable hydrophilic proteins. Subcellular localization analysis revealed that all 34 ARF transcription factor proteins in *P. euphratica* were localized to the nucleus, without transmembrane helix or signal peptide structure, indicating that PeARF proteins are nuclear localization proteins, which is consistent with previous studies concluding that ARF proteins are predominantly nuclear-localized [30,31,32,33]. Evolutionary analysis showed that *PeARF* gene family members could be divided into five subfamilies based on their evolutionary relationship, consistent with the results of studies on the peanut ARF gene family [34,35,36]. In the analysis of conserved domains and gene structures, we found that members on the same branch of the evolutionary tree exhibited higher conservation in gene and motif organization. However, variations in motif or exon–intron composition across different subfamilies have suggested structural rearrangements and fusions during evolution, warranting further investigation. Analysis of cis-acting elements revealed that the 2000 bp upstream promoter regions of *P. euphratica* ARF genes were enriched with hormone-responsive elements, particularly those responsive to auxin. This is consistent with previous studies emphasizing the role of ARF proteins in auxin signaling [2,8]. The second most abundant cis-acting elements were responsive to external light conditions, indicating that the *P. euphratica* ARF transcription factor gene family not only regulates growth and development but also plays a crucial role in responding to external stress, which aligns with recent studies demonstrating the key roles of plant ARFs in environmental stress responses [37,38,39].

Numerous studies have demonstrated the critical role of the ARF transcription factor gene family in plant growth and development, including the normal formation of vascular tissue, cotyledon development, leaf aging, flower development, and fruit ripening [40,41,42]. Previous studies have shown that various *AtARF* genes are expressed across multiple tissues, including root, stem, leaf, flower, and fruit, where transcriptionally activating *ATARF* genes regulate processes such as embryonic development, hypocotyl elongation, vascular tissue formation, lateral root formation, flower organ development, and phototropism. Transcriptionally repressive *AtARF* genes have been implicated in the regulation of seed size, lateral organ dorsoventral polarity, and seed dormancy and germination, as well as root gravitropism [43,44]. Similar expression patterns have also been observed in non-model species such as cucumber, tomato, and apple [45,46,47]. Evolutionary analysis of ARF proteins from *P. euphratica* and *A*. *thaliana* allows the inference of putative expression patterns and regulatory roles of *PeARF* genes based on their evolutionary proximity to functionally characterized *AtARFs.*

Previous studies have demonstrated that in *A. thaliana*, *AtARF4* influences the abaxial identity and laminar growth of lateral organs by regulating the activity of KANADI gene family members, thereby affecting the occurrence of leaf shape [48]. Similarly, elevated auxin levels have been shown to result in narrow leaves in *P. euphratica* [49]. In this study, analysis of the expression patterns of the ARF gene family in *P. euphratica* revealed that 17 ARF genes, including *PeARF1*, *PeARF33*, and *PeARF9*, were upregulated during the cell growth process in heterophylly. This conclusion is consistent with previous finding that XM_011046822.1 (*PeARF24*) was upregulated during the formation of ovate leaves [50], further indicating that upregulation of ARF genes can lead to the production of narrow leaves. Among the 34 ARF genes in *P. euphratica*, *PeARF1* exhibits the highest expression, which may be closely related to the formation of heterophylly. However, the specific function and its role in the regulation of heterophylly still require further study.

In recent years, members of the ARF transcription factor gene family have been identified in various plants [14,30,35]. However, the biological functions and regulatory mechanisms of most ARF genes remain unclear, which necessitate further studies to elucidate the specific functions and mechanisms of each ARF gene in plant growth and development. Technologies such as CRISPR-Cas9 and single-cell sequencing are expected to be more widely used in this research area, enabling more precise regulation of plant growth and development by ARFs. Thereby, this will provide important theoretical references for further improving the growth and development conditions of *P. euphratica* and increasing the utilization efficiency of forest resources in the future.

## 5. Conclusions

In this study, we conducted a comprehensive bioinformatics analysis of ARF gene family in *P. euphratica* based on genomic data, encompassing its physicochemical properties, evolutionary relationships, conserved domains, gene structures, cis-acting elements, chromosome localization, and expression patterns. A total of 56 *P. euphratica* ARF proteins and 34 *P. euphratica* ARF genes were identified. The *P. euphratica* ARF proteins were classified into five subfamilies based on their evolutionary distances. They are all unstable hydrophilic proteins located in the nucleus, lacking transmembrane structures, with length ranging from 592 to 1131 amino acids, protein molecular weights ranging from 65,303.76 to 126,034.12 Da, and isoelectric points ranging from 5.35 to 9.02. Members of the same subfamily showed conservation in gene structure, motif structure, and number of protein motifs. Analysis of promoter cis-acting elements revealed the presence of cis-acting elements related to hormones, growth, and environmental responses in the upstream region of 34 *P. euphratica* ARF genes. Among the 34 PeARF genes in *P. euphratica*, 17 showed relatively higher expression levels across young heteromorphic leaf types, with PeARF1 displaying the overall highest transcript abundance. While this suggests a potential role of PeARF1 in heterophylly development, further investigation is required to determine whether its expression is specifically related to cell growth, division, or differentiation. These results provide a foundation for future studies on the regulatory functions of ARF genes in *P. euphratica* leaf development.

## Data Availability

Data download links are given in the Materials and Methods.

## Supplementary Materials

The following supporting information can be downloaded at: https://www.mdpi.com/article/10.3390/plants14081248/s1, Figure S1. Prediction of tertiary structure of members in *P. euphratica*; Figure S2. Conserved domains of members of the ARF gene family in *P. euphratica*; Figure S3. Multiple sequence alignment of the *P. euphratica* ARF gene family members; Table S1. Basic information on ARF family members in *P. euphratica*; Table S2. Prediction of secondary structure of ARF family members in *P. euphratica*; Table S3. Structural similarity comparison of ARF proteins in *P. euphratica*; Table S4. Prediction of subcellular localization of ARF family members in *P. euphratica*; Table S5. Frequency and proportion of hormone-, light-, and stress-responsive cis-acting elements in the promoter regions of PeARF genes.
