# Supplementary material for: Genome-Wide Identification and Analysis of Auxin Response Factor Transcription Factor Gene Family in Populus euphratica
Source: Plants (Basel). 2025 Apr 19;14(8):1248. doi: 10.3390/plants14081248 (PMC12030272; doi:10.3390/plants14081248)
Supplement: Supplementary file 1 [file plants-14-01248-s001.zip › plants-3543466-supplementary.pdf]

Supplementary Figures

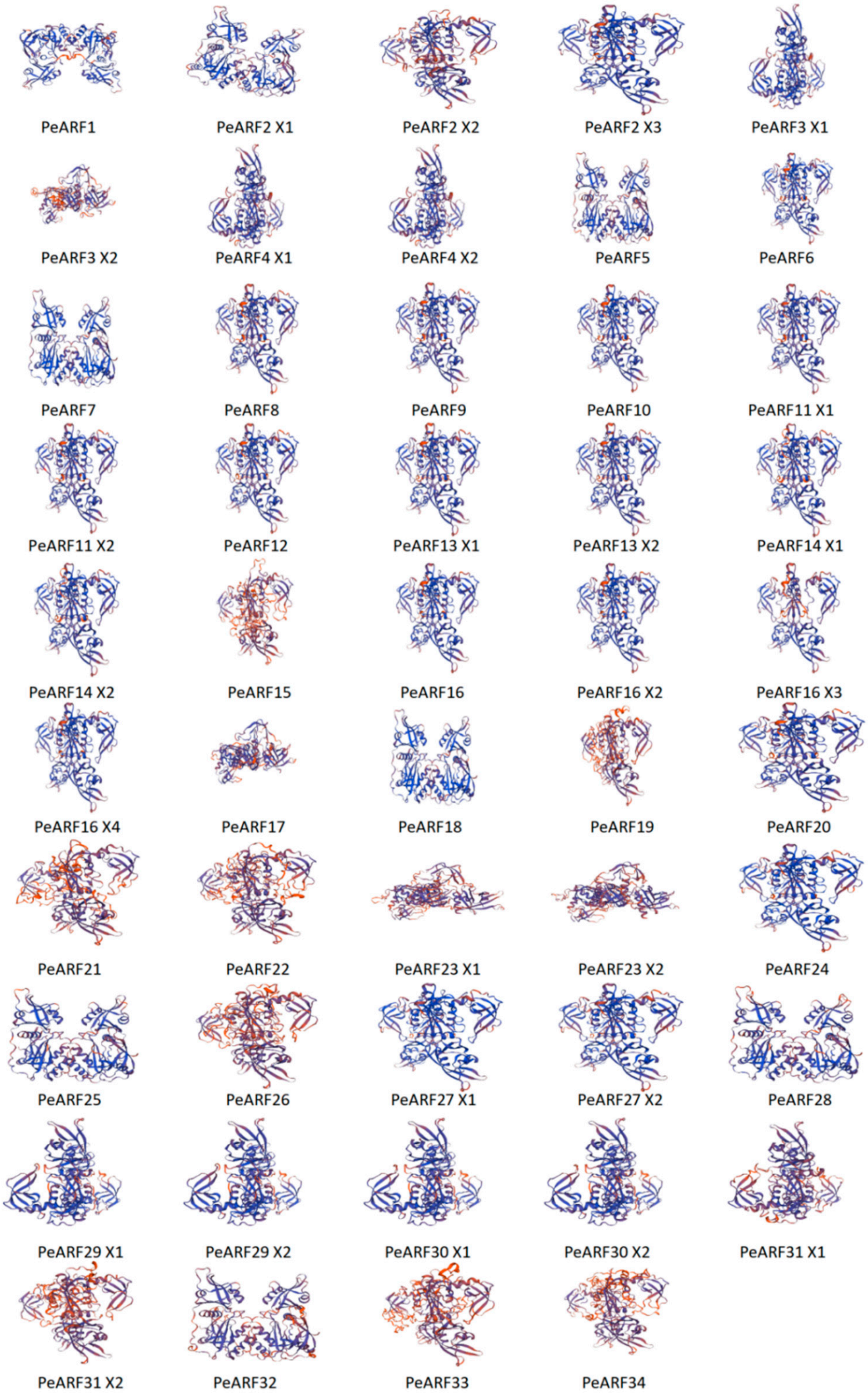

Figure S1. Prediction of tertiary structure of ARF family members in *P. euphratica.* Each panel represents a different ARF protein，in these diagrams, alpha-helices are typically shown as coiled ribbons, beta-sheets as arrows pointing in the direction of the beta-strands, and loops or turns as lines or ropes

connecting the secondary structures. The blue coloring of the ribbons likely indicates the backbone of the protein, while the orange and red elements could represent regions of functional significance.

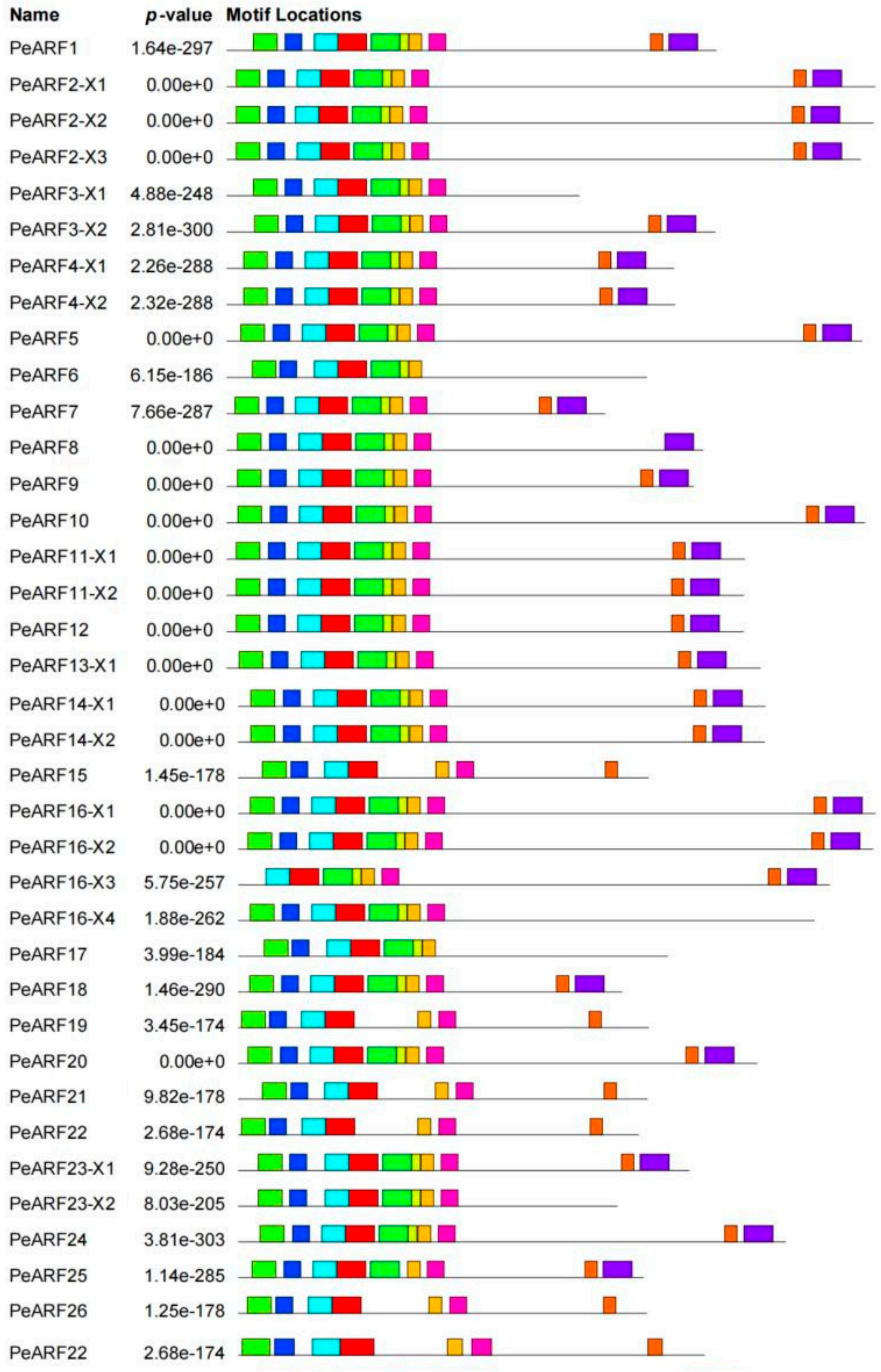

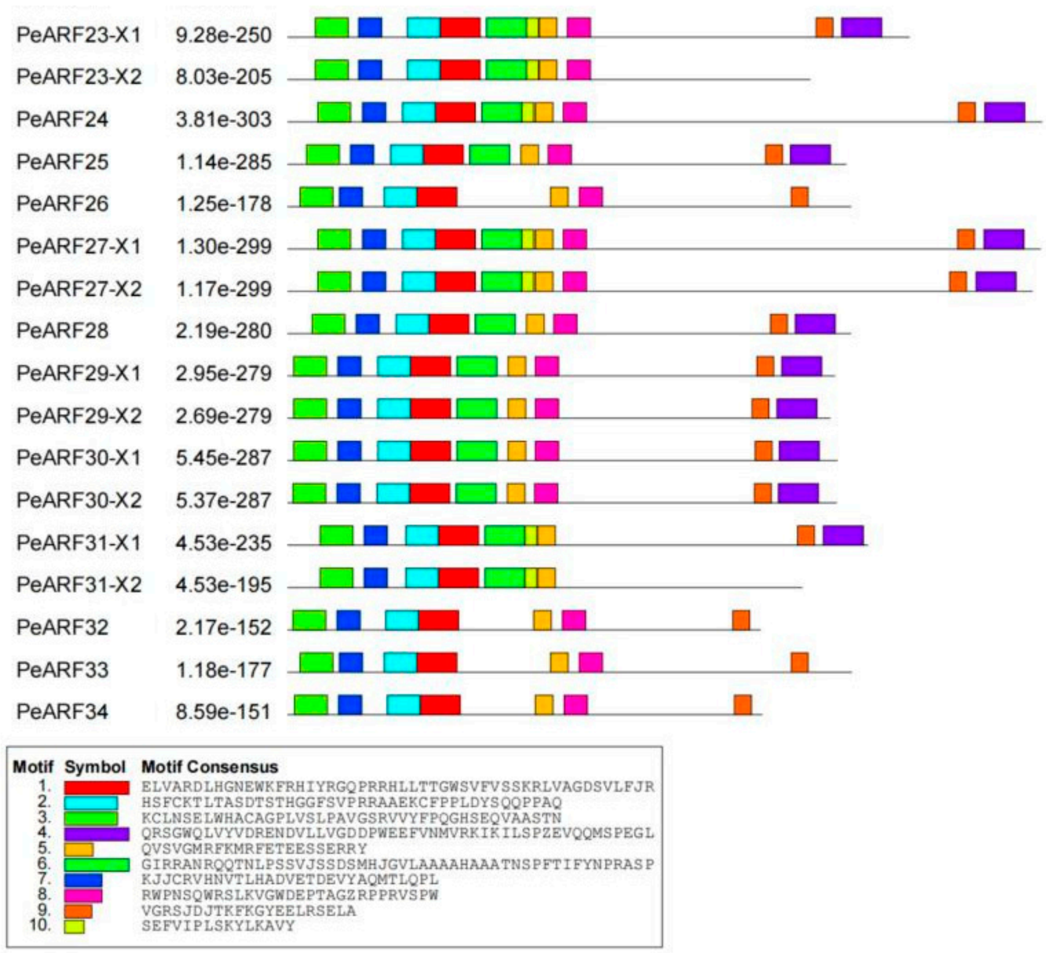

Figure S2. Conserved domains of members of the ARF gene family in *P. euphratica*. Different colors represent different domains (see legend), and the position and length of the bars reflect the specific position and size of each conserved domain in the protein sequence.

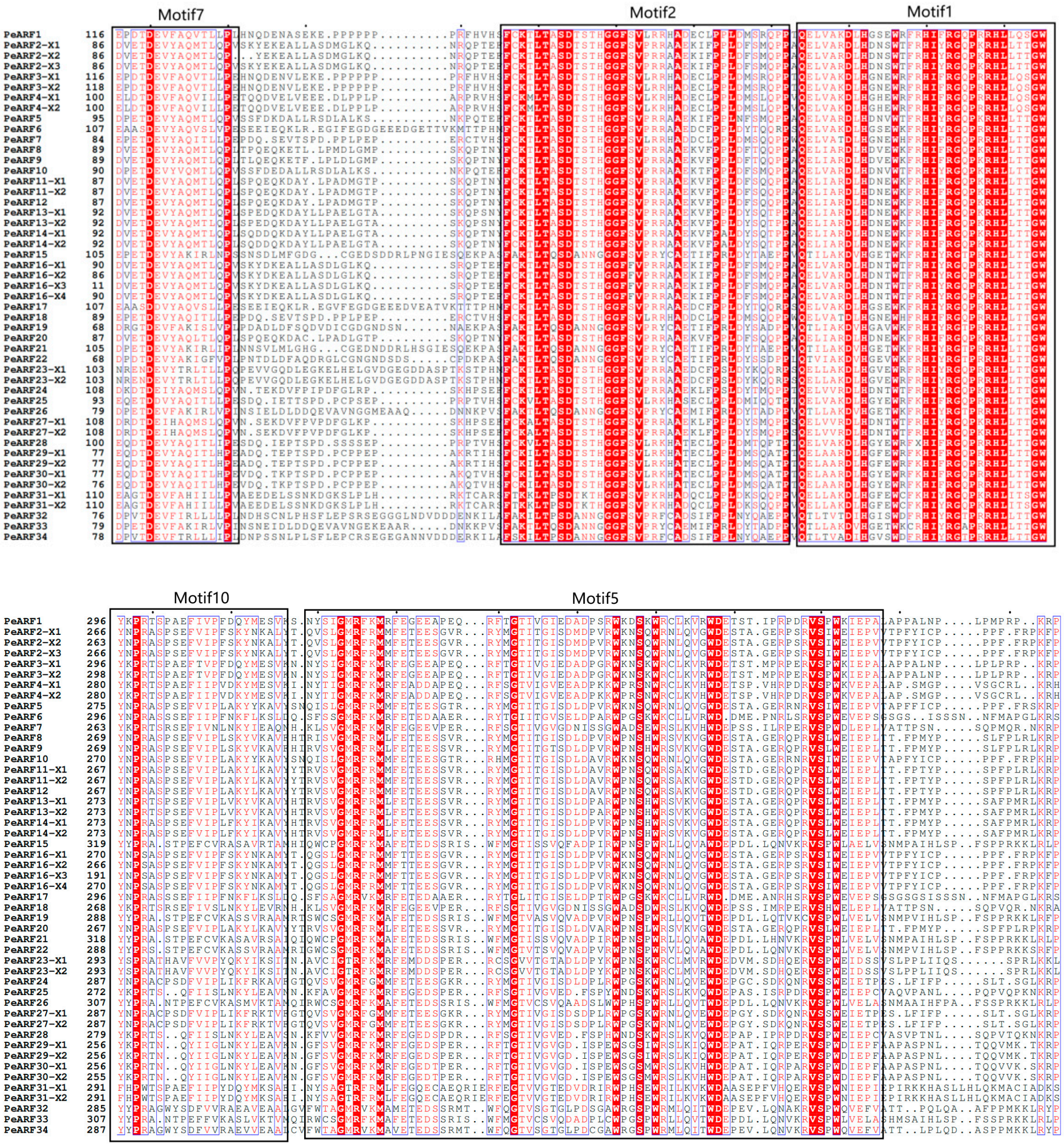

Figure S3. Multiple sequence alignment of the *P. euphratica* ARF gene family members. Conserved amino acids are marked in white text on a red background, while variable sites are shown in black text on a gray background.

Table S1. Basic information of ARF family members in *P. euphratica*

Gene	ID	Protein	Amino acid length/aa	molecular weight	Isoelectric Point (pH)
PeARF1	NW_011499959.1	PeARF1	854	94819.25	6.30
		PeARF2 X1	1131	126034.12	6.01
PeARF2	NW_011499934.1	PeARF2 X2	1128	125719.74	5.96
		PeARF2 X3	1106	123630.11	6.12

PeARF3	NW_011499937.1	PeARF3 X1	615	68374.63	6.77
		PeARF3 X2	852	94937.55	6.21
PeARF4	NW_011499855.1	PeARF4 X1	780	88463.01	6.68
		PeARF4 X2	782	88635.19	6.68
PeARF5	NW_011499854.1	PeARF5	1108	122073.78	6.28
PeARF6	NW_011500074.1	PeARF6	733	80505.78	6.28
PeARF7	NW_011500131.1	PeARF7	660	73506.44	5.84
PeARF8	NW_011500036.1	PeARF8	831	92659.33	5.98
PeARF9	NW_011499922.1	PeARF9	815	91313.86	5.96
PeARF10	NW_011499850.1	PeARF10	1113	123170.28	6.20
PeARF11	NW_011500020.1	PeARF11 X1	902	99793.27	6.23
		PeARF11 X2	902	99820.25	6.30
PeARF12	NW_011500844.1	PeARF12	904	99990.46	6.30
PeARF13	NW_011499869.1	PeARF13 X1	931	102777.01	6.01
		PeARF13 X2	930	102705.93	6.01
PeARF14	NW_011499892.1	PeARF14 X1	909	100409.78	6.17
		PeARF14 X2	908	100338.70	6.17
PeARF15	NW_011499888.1	PeARF15	708	78289.29	6.80
PeARF16	NW_011500217.1	PeARF16 X1	1099	123317.12	5.91
		PeARF16 X2	1095	122775.22	5.81
		PeARF16 X3	1020	114647.96	5.77
		PeARF16 X4	994	111621.17	6.37
PeARF17	NW_011499874.1	PeARF17	741	81669.96	6.28
PeARF18	NW_011499876.1	PeARF18	662	73935.30	5.99
PeARF19	NW_011499941.1	PeARF19	708	78023.47	9.02
PeARF20	NW_011500287.1	PeARF20	895	98996.97	6.21
PeARF21	NW_011499942.1	PeARF21	706	77624.71	8.23
PeARF22	NW_011500490.1	PeARF22	691	76791.97	7.23
PeARF23	NW_011499977.1	PeARF23 X1	778	86388.23	6.49

		PeARF23 X2	654	72542.65	8.51
PeARF24	NW_011499986.1	PeARF24	944	103506.21	5.35
PeARF25	NW_011499859.1	PeARF25	699	77852.79	6.23
PeARF26	NW_011500033.1	PeARF26	705	77649.28	6.63
PeARF27	NW_011499878.1	PeARF27 X1	942	103018.81	5.48
		PeARF27 X2	932	102094.83	5.53
PeARF28	NW_011499871.1	PeARF28	705	77969.87	5.95
PeARF29	NW_011499861.1	PeARF29 X1	685	76154.91	6.03
		PeARF29 X2	679	75543.21	6.03
PeARF30	NW_011499846.1	PeARF30 X1	688	76588.57	6.41
		PeARF30 X2	687	76517.49	6.41
PeARF31	NW_011499896.1	PeARF31 X1	726	81392.12	7.16
		PeARF31 X2	644	71998.40	8.29
PeARF32	NW_011500224.1	PeARF32	592	65303.76	5.55
PeARF33	NW_011500292.1	PeARF33	706	78405.15	7.88
PeARF34	NW_011499873.1	PeARF34	594	65604.10	5.77

Table S2. Prediction of Secondary Structure of ARF family members in *P. euphratica*

Protein	α-helix		Extended strand		β-sheet		Random coil	
	Amino acid length	Propotion	Amino acid length	Propotion	Amino acid length	Propotion	Amino acid length	Propotion
PeARF1	157	18.38%	134	14.52%	30	3.51%	543	65.58%
PeARF2 X1	313	27.67%	164	14.50%	91	8.05%	563	49.78%
PeARF2 X2	312	27.66%	164	14.54%	92	8.16%	560	49.65%
PeARF2 X3	292	26.40%	162	14.65%	91	8.23%	561	50.72%
PeARF3 X1	114	18.54%	100	16.26%	21	3.41%	380	61.79%
PeARF3 X2	164	19.25%	124	14.55%	37	4.34%	527	61.85%
PeARF4 X1	167	21.41%	124	15.90%	37	4.74%	452	57.95%
PeARF4 X2	164	20.97%	123	15.73%	35	4.48%	460	58.82%

PeARF5	332	29.96%	149	13.45%	55	4.96%	572	51.62%
PeARF6	115	15.69%	104	14.19%	31	4.23%	483	65.89%
PeARF7	125	18.94%	105	15.91%	32	4.85%	398	60.30%
PeARF8	241	29.00%	134	16.13%	44	5.29%	412	49.58%
PeARF9	229	28.10%	115	14.11%	35	4.29%	436	53.50%
PeARF10	340	30.55%	157	14.11%	69	6.20%	547	49.15%
PeARF11 X1	237	26.22%	138	15.27%	35	3.87%	494	54.65%
PeARF11 X2	230	25.50%	130	14.41%	32	3.55%	510	56.54%
PeARF12	239	26.50%	139	15.41%	31	3.44%	493	54.66%
PeARF13 X1	250	26.85%	126	13.53%	58	6.23%	497	53.38%
PeARF13 X2	250	26.88%	129	13.87%	67	7.20%	484	52.04%
PeARF14 X1	221	24.31%	124	13.64%	28	3.08%	536	58.97%
PeARF14 X2	136	19.21%	113	15.96%	34	4.80%	425	60.03%
PeARF15	136	19.21%	113	15.96%	34	4.80%	425	60.03%
PeARF16 X1	322	29.30%	194	17.65%	91	8.28%	492	44.77%
PeARF16 X2	315	28.77%	188	17.17%	92	8.40%	500	45.66%
PeARF16 X3	291	28.53%	174	17.06%	87	8.53%	468	45.88%
PeARF16 X4	303	30.48%	151	15.19%	65	6.54%	475	47.79%
PeARF17	113	15.25%	103	13.90%	27	3.64%	498	67.21%
PeARF18	139	21.00%	110	16.62%	32	4.83%	381	57.55%
PeARF19	111	15.68%	115	16.24%	32	4.52%	450	63.56%
PeARF20	241	26.93%	138	15.42%	43	4.80%	473	52.85%
PeARF21	136	19.26%	99	14.02%	32	4.53%	439	62.18%
PeARF22	126	18.23%	103	14.91%	33	4.78%	429	62.08%
PeARF23 X1	114	15.90%	114	15.90%	34	4.74%	455	63.46%
PeARF23 X2	88	13.46%	97	14.83%	23	3.52%	446	68.20%
PeARF24	258	27.33%	141	14.94%	39	4.13%	506	53.60%
PeARF25	134	19.17%	112	16.02%	30	4.29%	423	60.52%
PeARF26	138	19.57%	103	14.61%	35	4.96%	429	60.85%

PeARF27 X1	269	28.56%	162	17.20%	43	4.56%	468	49.68%
PeARF27 X2	282	30.26%	145	15.56%	45	4.83%	460	49.36%
PeARF28	123	17.45%	128	18.16%	32	4.54%	422	59.86%
PeARF29 X1	131	19.12%	117	17.08%	34	4.96%	403	58.83%
PeARF29 X2	135	19.88%	114	16.79%	33	4.86%	397	58.47%
PeARF30 X1	131	19.04%	117	17.01%	33	4.80%	407	59.16%
PeARF30 X2	122	17.76%	111	16.16%	33	4.80%	421	61.28%
PeARF31 X1	124	17.08%	105	14.46%	37	5.10%	460	63.36%
PeARF31 X2	86	13.35%	107	16.61%	27	4.19%	424	65.84%
PeARF32	96	16.22%	98	16.55%	22	3.72%	376	63.51%
PeARF33	132	18.70%	108	15.30%	30	4.25%	436	61.76%
PeARF34	107	18.01%	94	15.82%	23	3.87%	370	62.29%

Table S3. Structural similarity comparison of ARF proteins in *P. euphratica*

Protein 1 ID	Protein 2 ID	TM-score	RMSD	Seq. Identity (%)
PeARF01	PeARF02-X1	0.871	2.6	75.58
PeARF01	PeARF02-X2	0.932	1.25	30.33
PeARF01	PeARF02-X3	0.916	2.11	93.23
PeARF01	PeARF03-X1	0.845	1.11	49.01
PeARF01	PeARF03-X2	0.685	2.15	35.94
PeARF01	PeARF04-X1	0.842	3.23	90.99
PeARF01	PeARF04-X2	0.813	2.69	30.36
PeARF01	PeARF05	0.783	3.47	92.61
PeARF01	PeARF06	0.929	3.12	94.35
PeARF01	PeARF07	0.815	1.34	82.99
PeARF01	PeARF08	0.832	2.85	56.88
PeARF01	PeARF09	0.742	1.97	93.11
PeARF01	PeARF10	0.752	2.41	72.92

PeARF01	PeARF11-X1	0.706	3.43	63.49
PeARF01	PeARF11-X2	0.678	2.68	78.6
PeARF01	PeARF12	0.696	1.63	46.14
PeARF01	PeARF13-X1	0.7	2.78	83.75
PeARF01	PeARF13-X2	0.723	2.79	36.73
PeARF01	PeARF14-X1	0.789	1.1	68.23
PeARF01	PeARF14-X2	0.79	3.34	69.34
PeARF01	PeARF15	0.885	1.01	32.51
PeARF01	PeARF16-X1	0.811	1.21	30.2
PeARF01	PeARF16-X2	0.69	2.94	40.92
PeARF01	PeARF16-X3	0.73	2.22	34.91
PeARF01	PeARF16-X4	0.822	2.42	83.49
PeARF01	PeARF17	0.889	2.59	62.55
PeARF01	PeARF18	0.681	3.2	68.84
PeARF01	PeARF19	0.719	1.96	61.98
PeARF01	PeARF20	0.842	1.69	82.05
PeARF01	PeARF21	0.858	2.89	45.72
PeARF01	PeARF22	0.686	2.62	56
PeARF01	PeARF23-X1	0.809	1.32	46.89
PeARF01	PeARF23-X2	0.774	1.57	51.58
PeARF01	PeARF24	0.903	1.28	66.55
PeARF01	PeARF25	0.701	2.39	53.62
PeARF01	PeARF26	0.9	2.22	85.24
PeARF01	PeARF27-X1	0.766	1.26	61.74
PeARF01	PeARF27-X2	0.861	3.11	91.34
PeARF01	PeARF28	0.707	3.11	51.52

PeARF01	PeARF29-X1	0.794	1.9	79.78
PeARF01	PeARF29-X2	0.914	2.49	75.97
PeARF01	PeARF30-X1	0.916	1.64	58.98
PeARF01	PeARF30-X2	0.908	2.78	35.48
PeARF01	PeARF31-X1	0.745	1.78	71.47
PeARF01	PeARF31-X2	0.944	1.81	56.2
PeARF01	PeARF32	0.78	1.31	44.18
PeARF01	PeARF33	0.814	1.11	36.2
PeARF01	PeARF34	0.83	3.22	37.68
PeARF02-X1	PeARF02-X2	0.809	2.54	53.19
PeARF02-X1	PeARF02-X3	0.837	2.5	48.95
PeARF02-X1	PeARF03-X1	0.924	1.41	84.32
PeARF02-X1	PeARF03-X2	0.94	3.47	37.89
PeARF02-X1	PeARF04-X1	0.805	1.17	79.84
PeARF02-X1	PeARF04-X2	0.727	3.32	54.83
PeARF02-X1	PeARF05	0.668	2.48	49.92
PeARF02-X1	PeARF06	0.868	1.06	94.39
PeARF02-X1	PeARF07	0.673	1.18	73.99
PeARF02-X1	PeARF08	0.784	3.17	34
PeARF02-X1	PeARF09	0.907	1.57	47.11
PeARF02-X1	PeARF10	0.701	3.45	70.56
PeARF02-X1	PeARF11-X1	0.811	1.7	43.88
PeARF02-X1	PeARF11-X2	0.925	2.34	31.41
PeARF02-X1	PeARF12	0.92	2.97	31.63
PeARF02-X1	PeARF13-X1	0.875	2.28	65
PeARF02-X1	PeARF13-X2	0.859	1.37	53.79

PeARF02-X1	PeARF14-X1	0.779	3.09	40.42
PeARF02-X1	PeARF14-X2	0.824	2.72	32.19
PeARF02-X1	PeARF15	0.949	2.57	90.37
PeARF02-X1	PeARF16-X1	0.768	2.86	47.95
PeARF02-X1	PeARF16-X2	0.843	1.15	38.76
PeARF02-X1	PeARF16-X3	0.805	2.25	77.03
PeARF02-X1	PeARF16-X4	0.754	1.55	51.92
PeARF02-X1	PeARF17	0.901	1.28	80.03
PeARF02-X1	PeARF18	0.871	2.83	58.99
PeARF02-X1	PeARF19	0.816	2.22	52.5
PeARF02-X1	PeARF20	0.945	1.42	48.58
PeARF02-X1	PeARF21	0.944	2.88	53.51
PeARF02-X1	PeARF22	0.657	2.91	78.3
PeARF02-X1	PeARF23-X1	0.895	1.5	33.87
PeARF02-X1	PeARF23-X2	0.728	1.97	60.56
PeARF02-X1	PeARF24	0.894	2.48	32.73
PeARF02-X1	PeARF25	0.734	1.44	71.12
PeARF02-X1	PeARF26	0.872	1.38	79.87
PeARF02-X1	PeARF27-X1	0.829	2.09	87.9
PeARF02-X1	PeARF27-X2	0.861	1.59	39.51
PeARF02-X1	PeARF28	0.805	1.47	68.29
PeARF02-X1	PeARF29-X1	0.676	1.63	58.69
PeARF02-X1	PeARF29-X2	0.705	2.42	41.03
PeARF02-X1	PeARF30-X1	0.861	1.69	71.89
PeARF02-X1	PeARF30-X2	0.912	2.55	87.97
PeARF02-X1	PeARF31-X1	0.805	1.39	40.44

PeARF02-X1	PeARF31-X2	0.828	2.86	58.96
PeARF02-X1	PeARF32	0.719	1.4	61.85
PeARF02-X1	PeARF33	0.817	2.82	55.36
PeARF02-X1	PeARF34	0.701	3.04	32.62
PeARF02-X2	PeARF02-X3	0.89	1.38	75.97
PeARF02-X2	PeARF03-X1	0.742	2.47	33.73
PeARF02-X2	PeARF03-X2	0.679	1.18	74.83
PeARF02-X2	PeARF04-X1	0.777	2.84	44.13
PeARF02-X2	PeARF04-X2	0.754	2.21	32.37
PeARF02-X2	PeARF05	0.735	2.22	39.85
PeARF02-X2	PeARF06	0.653	1.45	32.83
PeARF02-X2	PeARF07	0.737	2.6	78.07
PeARF02-X2	PeARF08	0.771	2.73	62.59
PeARF02-X2	PeARF09	0.748	1.66	74.02
PeARF02-X2	PeARF10	0.785	3.29	58.71
PeARF02-X2	PeARF11-X1	0.862	1.91	77
PeARF02-X2	PeARF11-X2	0.652	1.52	53.9
PeARF02-X2	PeARF12	0.789	2.64	76.97
PeARF02-X2	PeARF13-X1	0.698	2.77	89.02
PeARF02-X2	PeARF13-X2	0.91	1.33	32.14
PeARF02-X2	PeARF14-X1	0.765	1.12	38.77
PeARF02-X2	PeARF14-X2	0.857	3.41	35.95
PeARF02-X2	PeARF15	0.9	1.18	42.77
PeARF02-X2	PeARF16-X1	0.695	2.34	75.27
PeARF02-X2	PeARF16-X2	0.808	1.61	51.77
PeARF02-X2	PeARF16-X3	0.95	3.37	35.37

PeARF02-X2	PeARF16-X4	0.756	2.72	67.28
PeARF02-X2	PeARF17	0.789	1.31	48.85
PeARF02-X2	PeARF18	0.757	1.58	63.13
PeARF02-X2	PeARF19	0.862	1.17	49.79
PeARF02-X2	PeARF20	0.717	3.01	41.68
PeARF02-X2	PeARF21	0.825	1.75	37.83
PeARF02-X2	PeARF22	0.882	2.41	72.58
PeARF02-X2	PeARF23-X1	0.765	2.98	38.32
PeARF02-X2	PeARF23-X2	0.824	1.34	77.82
PeARF02-X2	PeARF24	0.81	3.48	72.43
PeARF02-X2	PeARF25	0.712	1.57	81.45
PeARF02-X2	PeARF26	0.919	2.42	42.06
PeARF02-X2	PeARF27-X1	0.926	2.59	89.53
PeARF02-X2	PeARF27-X2	0.841	2.23	59.69
PeARF02-X2	PeARF28	0.836	3.21	52.84
PeARF02-X2	PeARF29-X1	0.92	3.37	73.73
PeARF02-X2	PeARF29-X2	0.73	2.16	30.75
PeARF02-X2	PeARF30-X1	0.93	3.06	71.28
PeARF02-X2	PeARF30-X2	0.889	2.15	45.9
PeARF02-X2	PeARF31-X1	0.891	2.32	79.61
PeARF02-X2	PeARF31-X2	0.72	3.06	62.43
PeARF02-X2	PeARF32	0.715	2.71	32.33
PeARF02-X2	PeARF33	0.872	1.76	61.83
PeARF02-X2	PeARF34	0.944	3.12	72.48
PeARF02-X3	PeARF03-X1	0.733	1.54	62.3
PeARF02-X3	PeARF03-X2	0.872	2.16	90.65

PeARF02-X3	PeARF04-X1	0.905	3.36	76.44
PeARF02-X3	PeARF04-X2	0.789	3.41	88.34
PeARF02-X3	PeARF05	0.695	3.07	47.31
PeARF02-X3	PeARF06	0.756	3.03	37.67
PeARF02-X3	PeARF07	0.725	1.39	68.67
PeARF02-X3	PeARF08	0.849	3.4	61.65
PeARF02-X3	PeARF09	0.755	1.8	66.57
PeARF02-X3	PeARF10	0.931	1.56	40.62
PeARF02-X3	PeARF11-X1	0.736	1.57	53.41
PeARF02-X3	PeARF11-X2	0.926	3.36	68.53
PeARF02-X3	PeARF12	0.742	1.12	32.59
PeARF02-X3	PeARF13-X1	0.906	3.45	83.85
PeARF02-X3	PeARF13-X2	0.945	2.67	88.43
PeARF02-X3	PeARF14-X1	0.697	3.32	66.81
PeARF02-X3	PeARF14-X2	0.781	1.24	65.75
PeARF02-X3	PeARF15	0.792	2	90.28
PeARF02-X3	PeARF16-X1	0.823	1.85	47.07
PeARF02-X3	PeARF16-X2	0.926	1.5	46.5
PeARF02-X3	PeARF16-X3	0.801	2.96	52.37
PeARF02-X3	PeARF16-X4	0.681	1.33	33.5
PeARF02-X3	PeARF17	0.702	1.51	31.61
PeARF02-X3	PeARF18	0.742	2.44	75.79
PeARF02-X3	PeARF19	0.746	1.61	38.18
PeARF02-X3	PeARF20	0.88	3.19	79.73
PeARF02-X3	PeARF21	0.731	2.95	37.14
PeARF02-X3	PeARF22	0.675	1.32	58.94

PeARF02-X3	PeARF23-X1	0.927	2.31	93.44
PeARF02-X3	PeARF23-X2	0.754	3.32	53.49
PeARF02-X3	PeARF24	0.846	2.25	63.07
PeARF02-X3	PeARF25	0.651	2.15	89.76
PeARF02-X3	PeARF26	0.822	2.08	48.23
PeARF02-X3	PeARF27-X1	0.709	1.78	81.37
PeARF02-X3	PeARF27-X2	0.659	3.41	78.62
PeARF02-X3	PeARF28	0.912	1.02	43.47
PeARF02-X3	PeARF29-X1	0.906	2.43	88.09
PeARF02-X3	PeARF29-X2	0.863	1.07	90.88
PeARF02-X3	PeARF30-X1	0.73	1.27	30.29
PeARF02-X3	PeARF30-X2	0.807	1.88	79.94
PeARF02-X3	PeARF31-X1	0.849	2.19	81.03
PeARF02-X3	PeARF31-X2	0.783	3.11	71.78
PeARF02-X3	PeARF32	0.833	2.85	85.66
PeARF02-X3	PeARF33	0.887	1.92	74.04
PeARF02-X3	PeARF34	0.707	1.61	44.63
PeARF03-X1	PeARF03-X2	0.732	1.12	66.12
PeARF03-X1	PeARF04-X1	0.709	1.83	42.19
PeARF03-X1	PeARF04-X2	0.853	1.66	89.96
PeARF03-X1	PeARF05	0.732	1.72	33.97
PeARF03-X1	PeARF06	0.746	1.78	84.44
PeARF03-X1	PeARF07	0.95	1.84	67.07
PeARF03-X1	PeARF08	0.819	2.2	58.99
PeARF03-X1	PeARF09	0.745	2.33	92.95
PeARF03-X1	PeARF10	0.672	3.07	80.83

PeARF03-X1	PeARF11-X1	0.814	2.45	87.8
PeARF03-X1	PeARF11-X2	0.748	1.93	74.52
PeARF03-X1	PeARF12	0.737	3.4	65.26
PeARF03-X1	PeARF13-X1	0.808	1.01	87.2
PeARF03-X1	PeARF13-X2	0.867	1.99	36.1
PeARF03-X1	PeARF14-X1	0.682	1.69	66.83
PeARF03-X1	PeARF14-X2	0.746	2.68	43.23
PeARF03-X1	PeARF15	0.801	2.61	82.22
PeARF03-X1	PeARF16-X1	0.731	3.38	51.55
PeARF03-X1	PeARF16-X2	0.724	2.85	86.63
PeARF03-X1	PeARF16-X3	0.685	1.82	34.01
PeARF03-X1	PeARF16-X4	0.82	2.92	84.92
PeARF03-X1	PeARF17	0.92	1.2	55.71
PeARF03-X1	PeARF18	0.796	3.05	62.69
PeARF03-X1	PeARF19	0.772	1.17	81.91
PeARF03-X1	PeARF20	0.689	1.21	39.22
PeARF03-X1	PeARF21	0.863	1.32	58.07
PeARF03-X1	PeARF22	0.816	1.6	41.76
PeARF03-X1	PeARF23-X1	0.778	2.92	41.5
PeARF03-X1	PeARF23-X2	0.766	2.69	46.56
PeARF03-X1	PeARF24	0.718	2.32	32.06
PeARF03-X1	PeARF25	0.763	2.92	72.21
PeARF03-X1	PeARF26	0.754	2.91	81.32
PeARF03-X1	PeARF27-X1	0.724	1.11	72.38
PeARF03-X1	PeARF27-X2	0.835	1.83	69.13
PeARF03-X1	PeARF28	0.683	2.9	56.84

PeARF03-X1	PeARF29-X1	0.934	1.55	69.13
PeARF03-X1	PeARF29-X2	0.838	1.06	37.67
PeARF03-X1	PeARF30-X1	0.886	3.01	33.08
PeARF03-X1	PeARF30-X2	0.668	1.76	85.46
PeARF03-X1	PeARF31-X1	0.705	1.15	43.28
PeARF03-X1	PeARF31-X2	0.739	1.03	94.75
PeARF03-X1	PeARF32	0.902	1.76	83.18
PeARF03-X1	PeARF33	0.765	1.68	38.63
PeARF03-X1	PeARF34	0.89	2.84	58.83
PeARF03-X2	PeARF04-X1	0.853	1.71	55.92
PeARF03-X2	PeARF04-X2	0.941	2.73	82.97
PeARF03-X2	PeARF05	0.93	2.83	79.86
PeARF03-X2	PeARF06	0.759	2.5	30.51
PeARF03-X2	PeARF07	0.761	2.28	92.79
PeARF03-X2	PeARF08	0.758	3.14	53.44
PeARF03-X2	PeARF09	0.729	2.29	77.99
PeARF03-X2	PeARF10	0.732	3.44	67.54
PeARF03-X2	PeARF11-X1	0.911	1.58	77.44
PeARF03-X2	PeARF11-X2	0.67	3.29	86.64
PeARF03-X2	PeARF12	0.87	2.68	90.08
PeARF03-X2	PeARF13-X1	0.869	1.52	47.53
PeARF03-X2	PeARF13-X2	0.884	2.11	31.11
PeARF03-X2	PeARF14-X1	0.912	1.83	56.54
PeARF03-X2	PeARF14-X2	0.708	1.75	78.98
PeARF03-X2	PeARF15	0.762	1.79	31.28
PeARF03-X2	PeARF16-X1	0.862	3.01	82.79

PeARF03-X2	PeARF16-X2	0.937	2.27	82.65
PeARF03-X2	PeARF16-X3	0.74	3.31	88.74
PeARF03-X2	PeARF16-X4	0.692	2.09	37.14
PeARF03-X2	PeARF17	0.707	2.47	43.96
PeARF03-X2	PeARF18	0.751	1.95	73.54
PeARF03-X2	PeARF19	0.685	3.31	82.06
PeARF03-X2	PeARF20	0.832	1.23	78.86
PeARF03-X2	PeARF21	0.856	2.32	60.11
PeARF03-X2	PeARF22	0.867	2.84	41.46
PeARF03-X2	PeARF23-X1	0.855	2.92	94.57
PeARF03-X2	PeARF23-X2	0.813	3.18	46.52
PeARF03-X2	PeARF24	0.899	3.02	46.8
PeARF03-X2	PeARF25	0.76	1.68	30.42
PeARF03-X2	PeARF26	0.929	3.4	86.01
PeARF03-X2	PeARF27-X1	0.696	1.75	46.21
PeARF03-X2	PeARF27-X2	0.918	1.74	90.19
PeARF03-X2	PeARF28	0.947	2.82	35.46
PeARF03-X2	PeARF29-X1	0.757	1.74	92.52
PeARF03-X2	PeARF29-X2	0.753	3.13	36.89
PeARF03-X2	PeARF30-X1	0.827	1.62	84.45
PeARF03-X2	PeARF30-X2	0.903	2.6	89.19
PeARF03-X2	PeARF31-X1	0.788	2.37	88.84
PeARF03-X2	PeARF31-X2	0.929	1.29	33.36
PeARF03-X2	PeARF32	0.913	2.68	62.6
PeARF03-X2	PeARF33	0.892	2.85	62.42
PeARF03-X2	PeARF34	0.864	1.03	64.6

PeARF04-X1	PeARF04-X2	0.722	2.96	61.74
PeARF04-X1	PeARF05	0.876	3.26	61
PeARF04-X1	PeARF06	0.68	2.36	78.31
PeARF04-X1	PeARF07	0.675	1.91	50.51
PeARF04-X1	PeARF08	0.935	1.39	90.6
PeARF04-X1	PeARF09	0.78	3.49	70.94
PeARF04-X1	PeARF10	0.918	1.33	79.27
PeARF04-X1	PeARF11-X1	0.77	1.72	90.08
PeARF04-X1	PeARF11-X2	0.687	2.71	82.91
PeARF04-X1	PeARF12	0.701	3.49	39.14
PeARF04-X1	PeARF13-X1	0.874	1.93	44.88
PeARF04-X1	PeARF13-X2	0.801	3.3	48.9
PeARF04-X1	PeARF14-X1	0.665	1.94	76.41
PeARF04-X1	PeARF14-X2	0.818	3.04	40.95
PeARF04-X1	PeARF15	0.93	2.68	57.58
PeARF04-X1	PeARF16-X1	0.904	3.05	31.37
PeARF04-X1	PeARF16-X2	0.836	1.83	65.54
PeARF04-X1	PeARF16-X3	0.838	2.36	54.26
PeARF04-X1	PeARF16-X4	0.807	1.73	53.76
PeARF04-X1	PeARF17	0.827	2.12	37.2
PeARF04-X1	PeARF18	0.894	1.94	86.57
PeARF04-X1	PeARF19	0.938	2.2	66.37
PeARF04-X1	PeARF20	0.848	3.19	74.57
PeARF04-X1	PeARF21	0.786	2.68	69.52
PeARF04-X1	PeARF22	0.744	1.28	47.07
PeARF04-X1	PeARF23-X1	0.73	2.02	83.97

PeARF04-X1	PeARF23-X2	0.871	3.34	86.38
PeARF04-X1	PeARF24	0.941	2.39	67.3
PeARF04-X1	PeARF25	0.791	3.36	89.12
PeARF04-X1	PeARF26	0.709	2.23	32.59
PeARF04-X1	PeARF27-X1	0.761	2.96	60.02
PeARF04-X1	PeARF27-X2	0.814	1.74	43.08
PeARF04-X1	PeARF28	0.682	1.14	64.98
PeARF04-X1	PeARF29-X1	0.85	1.8	48.07
PeARF04-X1	PeARF29-X2	0.718	3.17	51.9
PeARF04-X1	PeARF30-X1	0.898	3.01	46.82
PeARF04-X1	PeARF30-X2	0.941	1.51	38.17
PeARF04-X1	PeARF31-X1	0.762	3.02	53.11
PeARF04-X1	PeARF31-X2	0.665	2.85	75.89
PeARF04-X1	PeARF32	0.944	2.63	37.33
PeARF04-X1	PeARF33	0.729	1.41	86.98
PeARF04-X1	PeARF34	0.728	2.19	88.19
PeARF04-X2	PeARF05	0.833	1.26	85.16
PeARF04-X2	PeARF06	0.772	3.37	63.04
PeARF04-X2	PeARF07	0.918	2.1	33.43
PeARF04-X2	PeARF08	0.693	1.53	48.09
PeARF04-X2	PeARF09	0.899	1.99	66.79
PeARF04-X2	PeARF10	0.77	2.67	56.31
PeARF04-X2	PeARF11-X1	0.896	1.55	73.98
PeARF04-X2	PeARF11-X2	0.742	1.17	88.39
PeARF04-X2	PeARF12	0.908	1.97	85.8
PeARF04-X2	PeARF13-X1	0.719	2.89	73.1

PeARF04-X2	PeARF13-X2	0.795	2.38	70.96
PeARF04-X2	PeARF14-X1	0.681	2.89	74.38
PeARF04-X2	PeARF14-X2	0.652	3.23	31.32
PeARF04-X2	PeARF15	0.845	3.46	38.61
PeARF04-X2	PeARF16-X1	0.857	2.96	81.8
PeARF04-X2	PeARF16-X2	0.828	3.05	71.15
PeARF04-X2	PeARF16-X3	0.827	2.82	92.03
PeARF04-X2	PeARF16-X4	0.784	2.62	63.14
PeARF04-X2	PeARF17	0.878	1.6	53.1
PeARF04-X2	PeARF18	0.789	3.13	86.03
PeARF04-X2	PeARF19	0.698	3.15	51.92
PeARF04-X2	PeARF20	0.717	3.11	93.2
PeARF04-X2	PeARF21	0.796	2.37	70.96
PeARF04-X2	PeARF22	0.87	1.57	35.43
PeARF04-X2	PeARF23-X1	0.856	1.6	43.28
PeARF04-X2	PeARF23-X2	0.761	1.15	88.84
PeARF04-X2	PeARF24	0.719	1.83	48.62
PeARF04-X2	PeARF25	0.732	1.41	66.03
PeARF04-X2	PeARF26	0.73	1.59	49.4
PeARF04-X2	PeARF27-X1	0.66	2.27	51.69
PeARF04-X2	PeARF27-X2	0.815	1.83	73.31
PeARF04-X2	PeARF28	0.737	2.61	79.4
PeARF04-X2	PeARF29-X1	0.926	1.85	80.81
PeARF04-X2	PeARF29-X2	0.942	2.83	67.97
PeARF04-X2	PeARF30-X1	0.853	3.04	77.66
PeARF04-X2	PeARF30-X2	0.945	3.18	31.21

PeARF04-X2	PeARF31-X1	0.849	1.78	60.88
PeARF04-X2	PeARF31-X2	0.761	3.16	66.27
PeARF04-X2	PeARF32	0.845	1.55	74.68
PeARF04-X2	PeARF33	0.674	2.13	79.51
PeARF04-X2	PeARF34	0.899	2.92	82.43
PeARF05	PeARF06	0.776	3.31	57.78
PeARF05	PeARF07	0.932	1.14	35.78
PeARF05	PeARF08	0.668	1.03	46.29
PeARF05	PeARF09	0.878	1.84	65.38
PeARF05	PeARF10	0.787	1.94	78.31
PeARF05	PeARF11-X1	0.685	2.07	37.76
PeARF05	PeARF11-X2	0.765	1.04	77.01
PeARF05	PeARF12	0.913	2.88	62.19
PeARF05	PeARF13-X1	0.935	1.8	63.44
PeARF05	PeARF13-X2	0.911	1.33	74.34
PeARF05	PeARF14-X1	0.751	2.15	71.34
PeARF05	PeARF14-X2	0.681	3.43	51.68
PeARF05	PeARF15	0.679	3.36	49.05
PeARF05	PeARF16-X1	0.871	1.6	42.19
PeARF05	PeARF16-X2	0.924	3.25	31.69
PeARF05	PeARF16-X3	0.703	2.88	94.97
PeARF05	PeARF16-X4	0.842	2.73	83.77
PeARF05	PeARF17	0.846	1.36	92.85
PeARF05	PeARF18	0.917	2.35	48.76
PeARF05	PeARF19	0.703	2.8	42.42
PeARF05	PeARF20	0.829	3.17	83.14

PeARF05	PeARF21	0.71	3.14	72.02
PeARF05	PeARF22	0.665	2.89	67.75
PeARF05	PeARF23-X1	0.749	2.16	54.07
PeARF05	PeARF23-X2	0.82	1.91	66.34
PeARF05	PeARF24	0.899	1.69	52.5
PeARF05	PeARF25	0.726	1.52	67.26
PeARF05	PeARF26	0.814	1.22	61.48
PeARF05	PeARF27-X1	0.868	2.15	83.74
PeARF05	PeARF27-X2	0.803	1.37	55.38
PeARF05	PeARF28	0.898	1	94.08
PeARF05	PeARF29-X1	0.771	1.85	68.98
PeARF05	PeARF29-X2	0.838	3.11	76.2
PeARF05	PeARF30-X1	0.737	1.93	61.65
PeARF05	PeARF30-X2	0.734	3.33	35.59
PeARF05	PeARF31-X1	0.697	1.31	34.13
PeARF05	PeARF31-X2	0.766	2.81	51.9
PeARF05	PeARF32	0.666	2.03	55.04
PeARF05	PeARF33	0.823	2.44	73.21
PeARF05	PeARF34	0.749	1.47	56.66
PeARF06	PeARF07	0.817	2.87	91.41
PeARF06	PeARF08	0.942	2.33	94.12
PeARF06	PeARF09	0.815	1.86	46.67
PeARF06	PeARF10	0.87	1.72	51.37
PeARF06	PeARF11-X1	0.738	1.82	83.12
PeARF06	PeARF11-X2	0.759	2.31	70.49
PeARF06	PeARF12	0.773	2.41	73.26

PeARF06	PeARF13-X1	0.739	1.59	85.93
PeARF06	PeARF13-X2	0.823	2.13	47.14
PeARF06	PeARF14-X1	0.72	1.47	77.08
PeARF06	PeARF14-X2	0.719	1.09	90.03
PeARF06	PeARF15	0.695	1.68	34.44
PeARF06	PeARF16-X1	0.74	1.48	38.75
PeARF06	PeARF16-X2	0.654	3.24	83.36
PeARF06	PeARF16-X3	0.884	2.48	72.95
PeARF06	PeARF16-X4	0.729	2.8	79.99
PeARF06	PeARF17	0.865	3.28	44.25
PeARF06	PeARF18	0.685	2.04	78.95
PeARF06	PeARF19	0.853	2.74	90.7
PeARF06	PeARF20	0.779	1.35	62.07
PeARF06	PeARF21	0.655	2.2	41.33
PeARF06	PeARF22	0.772	1.68	36.76
PeARF06	PeARF23-X1	0.721	2.97	90.88
PeARF06	PeARF23-X2	0.847	2.39	61.32
PeARF06	PeARF24	0.672	2.6	33.12
PeARF06	PeARF25	0.772	2.94	30.01
PeARF06	PeARF26	0.776	3.42	55.03
PeARF06	PeARF27-X1	0.82	1.72	60.1
PeARF06	PeARF27-X2	0.792	2.32	60.87
PeARF06	PeARF28	0.691	2.11	64.78
PeARF06	PeARF29-X1	0.866	1.42	31.42
PeARF06	PeARF29-X2	0.698	2.75	36.05
PeARF06	PeARF30-X1	0.863	2.38	73.87

PeARF06	PeARF30-X2	0.851	1.62	84.08
PeARF06	PeARF31-X1	0.667	3.15	54.89
PeARF06	PeARF31-X2	0.782	2.27	90.13
PeARF06	PeARF32	0.709	2.17	90.19
PeARF06	PeARF33	0.751	3.16	30.13
PeARF06	PeARF34	0.752	1.61	51.15
PeARF07	PeARF08	0.899	1.39	72.21
PeARF07	PeARF09	0.919	1.8	93.7
PeARF07	PeARF10	0.823	2.71	30.59
PeARF07	PeARF11-X1	0.88	2.92	82.77
PeARF07	PeARF11-X2	0.84	1.45	81.36
PeARF07	PeARF12	0.656	1.31	54.2
PeARF07	PeARF13-X1	0.798	2.67	88.37
PeARF07	PeARF13-X2	0.912	3.31	74
PeARF07	PeARF14-X1	0.938	2.5	75.87
PeARF07	PeARF14-X2	0.911	2.9	55.05
PeARF07	PeARF15	0.686	1.73	68.09
PeARF07	PeARF16-X1	0.893	1.16	43.31
PeARF07	PeARF16-X2	0.78	2.52	82.74
PeARF07	PeARF16-X3	0.856	3.24	79.47
PeARF07	PeARF16-X4	0.653	1.18	66.08
PeARF07	PeARF17	0.778	1.18	78.89
PeARF07	PeARF18	0.814	2.01	30.33
PeARF07	PeARF19	0.827	3.16	88.58
PeARF07	PeARF20	0.785	1.45	75.13
PeARF07	PeARF21	0.885	1.7	90.79

PeARF07	PeARF22	0.697	2.61	94.64
PeARF07	PeARF23-X1	0.892	2.11	43.71
PeARF07	PeARF23-X2	0.922	2.04	59.67
PeARF07	PeARF24	0.779	1.04	77.11
PeARF07	PeARF25	0.891	3.34	92.29
PeARF07	PeARF26	0.884	2.43	84.55
PeARF07	PeARF27-X1	0.743	1.52	66.27
PeARF07	PeARF27-X2	0.671	2.67	82.31
PeARF07	PeARF28	0.9	3.17	76.85
PeARF07	PeARF29-X1	0.798	2.29	52.89
PeARF07	PeARF29-X2	0.808	2.52	31.73
PeARF07	PeARF30-X1	0.894	2	83.19
PeARF07	PeARF30-X2	0.887	1.07	57.1
PeARF07	PeARF31-X1	0.759	3.23	93.84
PeARF07	PeARF31-X2	0.808	1.66	51.61
PeARF07	PeARF32	0.718	2.09	81.7
PeARF07	PeARF33	0.831	1.77	53.39
PeARF07	PeARF34	0.677	2.99	63.79
PeARF08	PeARF09	0.889	2.87	77.5
PeARF08	PeARF10	0.796	2.82	68.75
PeARF08	PeARF11-X1	0.93	1.71	91.54
PeARF08	PeARF11-X2	0.862	3.4	69.82
PeARF08	PeARF12	0.753	3.17	85.6
PeARF08	PeARF13-X1	0.825	2.97	52.26
PeARF08	PeARF13-X2	0.787	2.3	66.3
PeARF08	PeARF14-X1	0.703	2.05	79.85

PeARF08	PeARF14-X2	0.766	2.09	58.23
PeARF08	PeARF15	0.685	1.5	86.05
PeARF08	PeARF16-X1	0.938	1.86	49.59
PeARF08	PeARF16-X2	0.798	2.27	68.9
PeARF08	PeARF16-X3	0.766	2.36	36.55
PeARF08	PeARF16-X4	0.845	2.29	36.15
PeARF08	PeARF17	0.752	2.92	72.62
PeARF08	PeARF18	0.814	2.95	76.65
PeARF08	PeARF19	0.897	3.06	41.17
PeARF08	PeARF20	0.722	1.56	79.96
PeARF08	PeARF21	0.901	2.87	42.58
PeARF08	PeARF22	0.777	1.24	44.4
PeARF08	PeARF23-X1	0.906	2.29	53.86
PeARF08	PeARF23-X2	0.734	2.75	50.46
PeARF08	PeARF24	0.815	1.26	45.92
PeARF08	PeARF25	0.661	2	78.62
PeARF08	PeARF26	0.912	1.02	39.21
PeARF08	PeARF27-X1	0.94	3.38	60.86
PeARF08	PeARF27-X2	0.883	1.45	51.24
PeARF08	PeARF28	0.7	2.04	84.83
PeARF08	PeARF29-X1	0.69	1.45	82.01
PeARF08	PeARF29-X2	0.94	2.55	78.84
PeARF08	PeARF30-X1	0.883	1.78	40.49
PeARF08	PeARF30-X2	0.701	1.66	71.78
PeARF08	PeARF31-X1	0.935	2.49	94.3
PeARF08	PeARF31-X2	0.907	1.19	63.73

PeARF08	PeARF32	0.874	2.44	58.44
PeARF08	PeARF33	0.771	2.09	69.32
PeARF08	PeARF34	0.668	1.15	34.6
PeARF09	PeARF10	0.797	3.03	69.66
PeARF09	PeARF11-X1	0.671	2.5	49.24
PeARF09	PeARF11-X2	0.789	3.15	37.84
PeARF09	PeARF12	0.936	3.33	30.83
PeARF09	PeARF13-X1	0.933	3.36	81.03
PeARF09	PeARF13-X2	0.78	1.96	78.22
PeARF09	PeARF14-X1	0.758	2.05	31.16
PeARF09	PeARF14-X2	0.763	1.03	63.84
PeARF09	PeARF15	0.832	3.11	94.78
PeARF09	PeARF16-X1	0.676	3.17	35.38
PeARF09	PeARF16-X2	0.719	3.05	73.89
PeARF09	PeARF16-X3	0.882	1.38	89.39
PeARF09	PeARF16-X4	0.927	1.12	79.78
PeARF09	PeARF17	0.829	2.65	70.21
PeARF09	PeARF18	0.71	2.94	62.09
PeARF09	PeARF19	0.947	3.32	72.41
PeARF09	PeARF20	0.933	3.11	83.43
PeARF09	PeARF21	0.716	1.6	71.78
PeARF09	PeARF22	0.723	3.4	57.3
PeARF09	PeARF23-X1	0.837	1.16	70.79
PeARF09	PeARF23-X2	0.849	1.75	89.94
PeARF09	PeARF24	0.815	1.28	41.39
PeARF09	PeARF25	0.848	1.5	80.32

PeARF09	PeARF26	0.87	1.44	45.38
PeARF09	PeARF27-X1	0.708	3.15	66.66
PeARF09	PeARF27-X2	0.936	1.17	73.05
PeARF09	PeARF28	0.86	1.42	64.13
PeARF09	PeARF29-X1	0.672	3.23	76.47
PeARF09	PeARF29-X2	0.677	3.17	77.12
PeARF09	PeARF30-X1	0.732	1.21	31.62
PeARF09	PeARF30-X2	0.901	2.22	73.03
PeARF09	PeARF31-X1	0.888	2.11	50.54
PeARF09	PeARF31-X2	0.756	1.46	55.44
PeARF09	PeARF32	0.915	1.51	70.05
PeARF09	PeARF33	0.68	1.26	48.04
PeARF09	PeARF34	0.805	1.15	39.92
PeARF10	PeARF11-X1	0.754	1.94	82.38
PeARF10	PeARF11-X2	0.863	1.76	68.67
PeARF10	PeARF12	0.701	2.25	79.13
PeARF10	PeARF13-X1	0.875	1.1	74.3
PeARF10	PeARF13-X2	0.908	2.59	85.56
PeARF10	PeARF14-X1	0.702	1.43	60.22
PeARF10	PeARF14-X2	0.831	3.01	47.67
PeARF10	PeARF15	0.744	1.03	68.16
PeARF10	PeARF16-X1	0.705	1.49	60.79
PeARF10	PeARF16-X2	0.844	1.09	65.31
PeARF10	PeARF16-X3	0.786	2.97	78.74
PeARF10	PeARF16-X4	0.661	2.1	86.14
PeARF10	PeARF17	0.943	2.16	50.51

PeARF10	PeARF18	0.908	2.04	64.99
PeARF10	PeARF19	0.913	2.81	72.33
PeARF10	PeARF20	0.707	1.55	37.32
PeARF10	PeARF21	0.817	3.42	71.11
PeARF10	PeARF22	0.858	2.7	60.25
PeARF10	PeARF23-X1	0.91	2.49	67.97
PeARF10	PeARF23-X2	0.854	2.44	89.09
PeARF10	PeARF24	0.686	1.46	60.65
PeARF10	PeARF25	0.708	1.8	48.4
PeARF10	PeARF26	0.907	2.83	44.67
PeARF10	PeARF27-X1	0.748	1.24	78.7
PeARF10	PeARF27-X2	0.862	2.05	50.35
PeARF10	PeARF28	0.934	2.33	82.67
PeARF10	PeARF29-X1	0.742	3.31	59
PeARF10	PeARF29-X2	0.658	1.93	69.06
PeARF10	PeARF30-X1	0.653	2.7	77.22
PeARF10	PeARF30-X2	0.745	3.41	48.55
PeARF10	PeARF31-X1	0.856	2.71	82.3
PeARF10	PeARF31-X2	0.679	1.95	88.98
PeARF10	PeARF32	0.706	2.17	70.09
PeARF10	PeARF33	0.791	1.68	52.93
PeARF10	PeARF34	0.788	3.03	69.74
PeARF11-X1	PeARF11-X2	0.797	2.29	47.29
PeARF11-X1	PeARF12	0.662	1.05	92.44
PeARF11-X1	PeARF13-X1	0.748	3.37	51.76
PeARF11-X1	PeARF13-X2	0.87	3.37	68.89

PeARF11-X1	PeARF14-X1	0.844	1.31	69.66
PeARF11-X1	PeARF14-X2	0.856	1.24	54.36
PeARF11-X1	PeARF15	0.755	2.02	32.69
PeARF11-X1	PeARF16-X1	0.857	2.97	34.13
PeARF11-X1	PeARF16-X2	0.8	3.22	85.66
PeARF11-X1	PeARF16-X3	0.761	1.86	54.22
PeARF11-X1	PeARF16-X4	0.813	3.41	33.81
PeARF11-X1	PeARF17	0.693	2.69	38.25
PeARF11-X1	PeARF18	0.654	3.2	51.01
PeARF11-X1	PeARF19	0.663	2.68	31.14
PeARF11-X1	PeARF20	0.785	1.03	42.64
PeARF11-X1	PeARF21	0.716	2.8	85.68
PeARF11-X1	PeARF22	0.698	3.36	76.18
PeARF11-X1	PeARF23-X1	0.949	1.28	72.51
PeARF11-X1	PeARF23-X2	0.673	2.31	66.65
PeARF11-X1	PeARF24	0.729	3.48	56.48
PeARF11-X1	PeARF25	0.83	1.99	44.19
PeARF11-X1	PeARF26	0.752	1.21	61.48
PeARF11-X1	PeARF27-X1	0.866	1.33	36.13
PeARF11-X1	PeARF27-X2	0.947	2.06	49.01
PeARF11-X1	PeARF28	0.84	3.25	87
PeARF11-X1	PeARF29-X1	0.914	3.11	80.98
PeARF11-X1	PeARF29-X2	0.903	2.99	79.09
PeARF11-X1	PeARF30-X1	0.8	2.33	64.96
PeARF11-X1	PeARF30-X2	0.792	2.37	39.88
PeARF11-X1	PeARF31-X1	0.794	1.63	47.84

PeARF11-X1	PeARF31-X2	0.819	1.14	68.82
PeARF11-X1	PeARF32	0.761	2.09	71.14
PeARF11-X1	PeARF33	0.679	2.93	47.98
PeARF11-X1	PeARF34	0.789	2.14	52.22
PeARF11-X2	PeARF12	0.906	3.48	61.11
PeARF11-X2	PeARF13-X1	0.831	1.62	36.69
PeARF11-X2	PeARF13-X2	0.675	1.84	68.74
PeARF11-X2	PeARF14-X1	0.656	2.3	93.37
PeARF11-X2	PeARF14-X2	0.901	1.67	62.75
PeARF11-X2	PeARF15	0.736	2.31	55.64
PeARF11-X2	PeARF16-X1	0.671	1.54	49.09
PeARF11-X2	PeARF16-X2	0.798	1.83	37.14
PeARF11-X2	PeARF16-X3	0.913	3.27	85.44
PeARF11-X2	PeARF16-X4	0.741	2.35	37.86
PeARF11-X2	PeARF17	0.933	1.21	91.84
PeARF11-X2	PeARF18	0.89	1.04	37.27
PeARF11-X2	PeARF19	0.9	3.24	69.4
PeARF11-X2	PeARF20	0.684	3.27	56.89
PeARF11-X2	PeARF21	0.667	2.33	43.54
PeARF11-X2	PeARF22	0.689	2.87	40
PeARF11-X2	PeARF23-X1	0.857	3.32	47.8
PeARF11-X2	PeARF23-X2	0.75	1.14	33.81
PeARF11-X2	PeARF24	0.929	1.63	34.82
PeARF11-X2	PeARF25	0.682	1.43	84.92
PeARF11-X2	PeARF26	0.928	3.02	33.32
PeARF11-X2	PeARF27-X1	0.769	3.4	43.12

PeARF11-X2	PeARF27-X2	0.786	2.17	32.2
PeARF11-X2	PeARF28	0.741	3.44	60.74
PeARF11-X2	PeARF29-X1	0.893	3.03	74.94
PeARF11-X2	PeARF29-X2	0.772	2.21	91.23
PeARF11-X2	PeARF30-X1	0.818	2.59	46.76
PeARF11-X2	PeARF30-X2	0.756	3.02	75.09
PeARF11-X2	PeARF31-X1	0.823	2.59	49.18
PeARF11-X2	PeARF31-X2	0.901	2.04	48.86
PeARF11-X2	PeARF32	0.87	3.22	44.36
PeARF11-X2	PeARF33	0.912	2.77	58.96
PeARF11-X2	PeARF34	0.694	1.84	44.58
PeARF12	PeARF13-X1	0.896	1	47.49
PeARF12	PeARF13-X2	0.762	1.01	43.44
PeARF12	PeARF14-X1	0.918	2.53	52.15
PeARF12	PeARF14-X2	0.795	1.52	79.12
PeARF12	PeARF15	0.838	1.57	58.19
PeARF12	PeARF16-X1	0.907	2.78	94.52
PeARF12	PeARF16-X2	0.735	2.59	54.35
PeARF12	PeARF16-X3	0.69	2.62	61.33
PeARF12	PeARF16-X4	0.774	1.99	61.39
PeARF12	PeARF17	0.754	2.01	75.53
PeARF12	PeARF18	0.784	1.13	36.22
PeARF12	PeARF19	0.905	1.3	77.04
PeARF12	PeARF20	0.728	2.2	83.2
PeARF12	PeARF21	0.771	1.37	57.9
PeARF12	PeARF22	0.919	3.19	81.4

PeARF12	PeARF23-X1	0.704	2.29	44.44
PeARF12	PeARF23-X2	0.696	2.3	63.1
PeARF12	PeARF24	0.853	2.08	57.1
PeARF12	PeARF25	0.886	3.25	76.73
PeARF12	PeARF26	0.94	2.94	83.57
PeARF12	PeARF27-X1	0.67	2	58.61
PeARF12	PeARF27-X2	0.897	1.69	46.72
PeARF12	PeARF28	0.681	3.26	61.78
PeARF12	PeARF29-X1	0.651	1.75	57.9
PeARF12	PeARF29-X2	0.679	2.45	65.4
PeARF12	PeARF30-X1	0.651	2.65	77.48
PeARF12	PeARF30-X2	0.681	2.92	31.28
PeARF12	PeARF31-X1	0.945	2.45	54.64
PeARF12	PeARF31-X2	0.727	1.82	85.56
PeARF12	PeARF32	0.929	1.41	67.69
PeARF12	PeARF33	0.804	1.06	87.53
PeARF12	PeARF34	0.83	1.52	61.82
PeARF13-X1	PeARF13-X2	0.882	1.01	32.37
PeARF13-X1	PeARF14-X1	0.655	2.4	38.88
PeARF13-X1	PeARF14-X2	0.739	2.67	78.13
PeARF13-X1	PeARF15	0.735	2.46	85.52
PeARF13-X1	PeARF16-X1	0.673	3.37	67.21
PeARF13-X1	PeARF16-X2	0.862	1.86	39.21
PeARF13-X1	PeARF16-X3	0.943	3.46	39.43
PeARF13-X1	PeARF16-X4	0.677	2.91	68.32
PeARF13-X1	PeARF17	0.809	1.26	49.7

PeARF13-X1	PeARF18	0.85	3.25	76.54
PeARF13-X1	PeARF19	0.813	3.08	78.18
PeARF13-X1	PeARF20	0.874	2.7	37.92
PeARF13-X1	PeARF21	0.746	2.4	74.78
PeARF13-X1	PeARF22	0.91	1.45	67.18
PeARF13-X1	PeARF23-X1	0.875	1.84	56.21
PeARF13-X1	PeARF23-X2	0.94	1	37.76
PeARF13-X1	PeARF24	0.689	2.65	81.78
PeARF13-X1	PeARF25	0.937	3.16	94.98
PeARF13-X1	PeARF26	0.664	3.36	82.2
PeARF13-X1	PeARF27-X1	0.652	2.03	80.47
PeARF13-X1	PeARF27-X2	0.835	3.47	73.28
PeARF13-X1	PeARF28	0.822	1.22	39.27
PeARF13-X1	PeARF29-X1	0.834	1.88	89.29
PeARF13-X1	PeARF29-X2	0.854	2.87	38.34
PeARF13-X1	PeARF30-X1	0.79	3.46	74.54
PeARF13-X1	PeARF30-X2	0.764	2.55	35.62
PeARF13-X1	PeARF31-X1	0.674	2.85	76.35
PeARF13-X1	PeARF31-X2	0.668	3.3	82.2
PeARF13-X1	PeARF32	0.848	1.14	54.41
PeARF13-X1	PeARF33	0.65	1.17	30.45
PeARF13-X1	PeARF34	0.78	2.14	69.1
PeARF13-X2	PeARF14-X1	0.94	2.48	94.85
PeARF13-X2	PeARF14-X2	0.881	2.13	59.9
PeARF13-X2	PeARF15	0.815	2.13	90.52
PeARF13-X2	PeARF16-X1	0.865	1.43	55.65

PeARF13-X2	PeARF16-X2	0.851	2.69	86.05
PeARF13-X2	PeARF16-X3	0.905	1.48	58.83
PeARF13-X2	PeARF16-X4	0.878	2.48	59.05
PeARF13-X2	PeARF17	0.652	3.08	55.67
PeARF13-X2	PeARF18	0.793	2.44	77.92
PeARF13-X2	PeARF19	0.801	1.57	53.8
PeARF13-X2	PeARF20	0.894	2.72	44.18
PeARF13-X2	PeARF21	0.805	1.74	57.74
PeARF13-X2	PeARF22	0.91	3.25	63.35
PeARF13-X2	PeARF23-X1	0.944	1.75	55.19
PeARF13-X2	PeARF23-X2	0.829	2.46	68.99
PeARF13-X2	PeARF24	0.845	2.17	54.47
PeARF13-X2	PeARF25	0.754	3.02	46.53
PeARF13-X2	PeARF26	0.913	1.57	48.32
PeARF13-X2	PeARF27-X1	0.693	1.71	32.4
PeARF13-X2	PeARF27-X2	0.688	1.58	78.81
PeARF13-X2	PeARF28	0.722	2.19	39.59
PeARF13-X2	PeARF29-X1	0.659	1.75	32.36
PeARF13-X2	PeARF29-X2	0.722	1.31	70.69
PeARF13-X2	PeARF30-X1	0.657	3.19	87.56
PeARF13-X2	PeARF30-X2	0.855	2.77	33.28
PeARF13-X2	PeARF31-X1	0.807	1.91	92.57
PeARF13-X2	PeARF31-X2	0.71	2.94	41.02
PeARF13-X2	PeARF32	0.662	1.67	41.29
PeARF13-X2	PeARF33	0.748	1.75	71.73
PeARF13-X2	PeARF34	0.68	3.07	92.06

PeARF14-X1	PeARF14-X2	0.93	2.65	71.38
PeARF14-X1	PeARF15	0.896	1.42	82.8
PeARF14-X1	PeARF16-X1	0.829	1.07	41.58
PeARF14-X1	PeARF16-X2	0.721	1.19	36.57
PeARF14-X1	PeARF16-X3	0.783	2.15	68.44
PeARF14-X1	PeARF16-X4	0.717	1.77	55.03
PeARF14-X1	PeARF17	0.76	2.78	69.39
PeARF14-X1	PeARF18	0.886	1.53	40.24
PeARF14-X1	PeARF19	0.697	2.92	45.49
PeARF14-X1	PeARF20	0.703	2.57	56.6
PeARF14-X1	PeARF21	0.791	2.26	49.24
PeARF14-X1	PeARF22	0.863	3.36	94.52
PeARF14-X1	PeARF23-X1	0.78	2.39	42.69
PeARF14-X1	PeARF23-X2	0.754	1.13	42.61
PeARF14-X1	PeARF24	0.783	2.62	39.23
PeARF14-X1	PeARF25	0.894	1.54	80.67
PeARF14-X1	PeARF26	0.824	1.91	91.64
PeARF14-X1	PeARF27-X1	0.928	1.2	40.01
PeARF14-X1	PeARF27-X2	0.88	1.32	84.9
PeARF14-X1	PeARF28	0.857	1.19	82.24
PeARF14-X1	PeARF29-X1	0.903	1.4	86.07
PeARF14-X1	PeARF29-X2	0.714	1.36	42.43
PeARF14-X1	PeARF30-X1	0.885	2.77	35.34
PeARF14-X1	PeARF30-X2	0.937	2.01	35.47
PeARF14-X1	PeARF31-X1	0.803	2.21	71.13
PeARF14-X1	PeARF31-X2	0.85	1.28	47.76

PeARF14-X1	PeARF32	0.872	1.86	82.89
PeARF14-X1	PeARF33	0.87	1.23	66.8
PeARF14-X1	PeARF34	0.76	1.3	49.02
PeARF14-X2	PeARF15	0.898	3.2	64.2
PeARF14-X2	PeARF16-X1	0.838	2.32	71.89
PeARF14-X2	PeARF16-X2	0.915	1.61	34.4
PeARF14-X2	PeARF16-X3	0.732	1.59	78.51
PeARF14-X2	PeARF16-X4	0.704	2.57	35.55
PeARF14-X2	PeARF17	0.908	2.85	81.25
PeARF14-X2	PeARF18	0.652	2.14	87.3
PeARF14-X2	PeARF19	0.802	2.56	35.66
PeARF14-X2	PeARF20	0.82	2.02	64.23
PeARF14-X2	PeARF21	0.672	1.03	39.94
PeARF14-X2	PeARF22	0.814	2.47	43.44
PeARF14-X2	PeARF23-X1	0.698	1.79	82.62
PeARF14-X2	PeARF23-X2	0.93	2.92	45.35
PeARF14-X2	PeARF24	0.85	1.45	55.9
PeARF14-X2	PeARF25	0.743	1.49	51.2
PeARF14-X2	PeARF26	0.792	1.58	38.33
PeARF14-X2	PeARF27-X1	0.687	1.27	92.05
PeARF14-X2	PeARF27-X2	0.933	1.69	81.11
PeARF14-X2	PeARF28	0.769	2.51	48.38
PeARF14-X2	PeARF29-X1	0.869	2.64	91.67
PeARF14-X2	PeARF29-X2	0.867	2.45	53.77
PeARF14-X2	PeARF30-X1	0.802	2.02	76.24
PeARF14-X2	PeARF30-X2	0.83	1.49	93.33

PeARF14-X2	PeARF31-X1	0.696	2.72	71.45
PeARF14-X2	PeARF31-X2	0.656	1.34	58.88
PeARF14-X2	PeARF32	0.725	2.16	65.83
PeARF14-X2	PeARF33	0.826	2.64	66.83
PeARF14-X2	PeARF34	0.864	1.69	53.4
PeARF15	PeARF16-X1	0.863	2.36	82.93
PeARF15	PeARF16-X2	0.885	1.4	92.27
PeARF15	PeARF16-X3	0.783	2.27	86.79
PeARF15	PeARF16-X4	0.849	1.31	55.76
PeARF15	PeARF17	0.784	2.94	32.72
PeARF15	PeARF18	0.886	3.38	66.98
PeARF15	PeARF19	0.839	3.29	44.26
PeARF15	PeARF20	0.749	1.06	62.38
PeARF15	PeARF21	0.848	2.31	84.9
PeARF15	PeARF22	0.747	3.11	65.4
PeARF15	PeARF23-X1	0.812	2.79	48.16
PeARF15	PeARF23-X2	0.772	3.19	86.01
PeARF15	PeARF24	0.84	2.31	42.46
PeARF15	PeARF25	0.9	1.07	70.45
PeARF15	PeARF26	0.691	1.2	56.62
PeARF15	PeARF27-X1	0.747	2.23	45.71
PeARF15	PeARF27-X2	0.724	3.01	46.01
PeARF15	PeARF28	0.724	1.9	69.85
PeARF15	PeARF29-X1	0.705	1.78	49.03
PeARF15	PeARF29-X2	0.831	3.49	91.18
PeARF15	PeARF30-X1	0.917	1.21	84.04

PeARF15	PeARF30-X2	0.793	1.37	43.73
PeARF15	PeARF31-X1	0.873	1.42	86.37
PeARF15	PeARF31-X2	0.655	1.01	63.18
PeARF15	PeARF32	0.738	2.29	60.54
PeARF15	PeARF33	0.679	1.18	69.69
PeARF15	PeARF34	0.847	1.07	43.17
PeARF16-X1	PeARF16-X2	0.884	2.97	74.72
PeARF16-X1	PeARF16-X3	0.655	2.67	90.88
PeARF16-X1	PeARF16-X4	0.939	1.32	77.48
PeARF16-X1	PeARF17	0.846	1.54	74.92
PeARF16-X1	PeARF18	0.711	3.36	75.04
PeARF16-X1	PeARF19	0.742	3.21	33.13
PeARF16-X1	PeARF20	0.753	2.11	83.67
PeARF16-X1	PeARF21	0.818	2.75	76.19
PeARF16-X1	PeARF22	0.779	2.82	53.19
PeARF16-X1	PeARF23-X1	0.69	2.28	54.62
PeARF16-X1	PeARF23-X2	0.67	3.3	60.23
PeARF16-X1	PeARF24	0.668	3.18	59.81
PeARF16-X1	PeARF25	0.718	2.86	36.21
PeARF16-X1	PeARF26	0.838	1.28	58.69
PeARF16-X1	PeARF27-X1	0.93	1.31	31.03
PeARF16-X1	PeARF27-X2	0.926	2.99	47.53
PeARF16-X1	PeARF28	0.937	2.13	91.98
PeARF16-X1	PeARF29-X1	0.855	2.67	77.99
PeARF16-X1	PeARF29-X2	0.834	2.31	46.25
PeARF16-X1	PeARF30-X1	0.83	1.75	52.33

PeARF16-X1	PeARF30-X2	0.832	1.38	44.03
PeARF16-X1	PeARF31-X1	0.799	3.01	37.77
PeARF16-X1	PeARF31-X2	0.898	1.81	76.53
PeARF16-X1	PeARF32	0.7	2.19	30.21
PeARF16-X1	PeARF33	0.708	2.96	70.29
PeARF16-X1	PeARF34	0.789	3.29	69.9
PeARF16-X2	PeARF16-X3	0.747	3.34	82.08
PeARF16-X2	PeARF16-X4	0.917	1.1	58.06
PeARF16-X2	PeARF17	0.712	3.34	52.72
PeARF16-X2	PeARF18	0.876	1.7	83.62
PeARF16-X2	PeARF19	0.891	2.82	49.97
PeARF16-X2	PeARF20	0.714	2.25	44.47
PeARF16-X2	PeARF21	0.751	3.18	57.02
PeARF16-X2	PeARF22	0.827	2.8	82.09
PeARF16-X2	PeARF23-X1	0.761	1.14	57.53
PeARF16-X2	PeARF23-X2	0.676	2.66	65.86
PeARF16-X2	PeARF24	0.733	2.04	51.65
PeARF16-X2	PeARF25	0.794	2.43	72.26
PeARF16-X2	PeARF26	0.729	1.38	62.1
PeARF16-X2	PeARF27-X1	0.821	1.44	66.55
PeARF16-X2	PeARF27-X2	0.891	2.91	93.48
PeARF16-X2	PeARF28	0.718	3.34	42.06
PeARF16-X2	PeARF29-X1	0.862	2.05	71.48
PeARF16-X2	PeARF29-X2	0.67	1.16	61.11
PeARF16-X2	PeARF30-X1	0.761	1	78.33
PeARF16-X2	PeARF30-X2	0.719	1.85	33.72

PeARF16-X2	PeARF31-X1	0.851	3.46	90.01
PeARF16-X2	PeARF31-X2	0.707	1.77	78.92
PeARF16-X2	PeARF32	0.783	2.18	61.06
PeARF16-X2	PeARF33	0.776	2.07	34.39
PeARF16-X2	PeARF34	0.783	2.25	44.48
PeARF16-X3	PeARF16-X4	0.947	1.08	35.23
PeARF16-X3	PeARF17	0.884	2.66	64.92
PeARF16-X3	PeARF18	0.878	2.94	63.07
PeARF16-X3	PeARF19	0.684	3.35	81.46
PeARF16-X3	PeARF20	0.723	3.48	31.94
PeARF16-X3	PeARF21	0.652	3.42	34.48
PeARF16-X3	PeARF22	0.852	2.94	34.7
PeARF16-X3	PeARF23-X1	0.671	1.9	78.48
PeARF16-X3	PeARF23-X2	0.697	3.11	55.81
PeARF16-X3	PeARF24	0.679	1.48	35.29
PeARF16-X3	PeARF25	0.72	2.82	35.33
PeARF16-X3	PeARF26	0.752	2.33	72.96
PeARF16-X3	PeARF27-X1	0.789	1.57	41.42
PeARF16-X3	PeARF27-X2	0.876	2.52	55.19
PeARF16-X3	PeARF28	0.888	3.04	94.66
PeARF16-X3	PeARF29-X1	0.875	2.97	74.58
PeARF16-X3	PeARF29-X2	0.717	3.39	68.58
PeARF16-X3	PeARF30-X1	0.946	2.61	80.57
PeARF16-X3	PeARF30-X2	0.766	1.98	73.42
PeARF16-X3	PeARF31-X1	0.879	2.29	67.06
PeARF16-X3	PeARF31-X2	0.658	3.17	50.4

PeARF16-X3	PeARF32	0.893	2.82	36.52
PeARF16-X3	PeARF33	0.787	1.25	65.49
PeARF16-X3	PeARF34	0.769	1.02	79.32
PeARF16-X4	PeARF17	0.913	2.66	93.69
PeARF16-X4	PeARF18	0.927	1.15	65.45
PeARF16-X4	PeARF19	0.68	3.24	76.14
PeARF16-X4	PeARF20	0.832	3.18	47.06
PeARF16-X4	PeARF21	0.706	2.71	64.25
PeARF16-X4	PeARF22	0.831	2.2	69.4
PeARF16-X4	PeARF23-X1	0.787	3.43	91.16
PeARF16-X4	PeARF23-X2	0.807	1.78	48.71
PeARF16-X4	PeARF24	0.947	1.43	83.39
PeARF16-X4	PeARF25	0.94	1.21	48.06
PeARF16-X4	PeARF26	0.906	3.13	77.06
PeARF16-X4	PeARF27-X1	0.894	2.4	61.1
PeARF16-X4	PeARF27-X2	0.906	1.44	51.48
PeARF16-X4	PeARF28	0.741	2.93	82.72
PeARF16-X4	PeARF29-X1	0.913	1.44	55.7
PeARF16-X4	PeARF29-X2	0.704	3.12	48.72
PeARF16-X4	PeARF30-X1	0.698	1.47	81.57
PeARF16-X4	PeARF30-X2	0.749	1.41	65.64
PeARF16-X4	PeARF31-X1	0.738	2.76	79.26
PeARF16-X4	PeARF31-X2	0.874	3.45	81.53
PeARF16-X4	PeARF32	0.895	2.17	56.34
PeARF16-X4	PeARF33	0.755	2.75	36.04
PeARF16-X4	PeARF34	0.85	2.19	86.18

PeARF17	PeARF18	0.926	2.09	85.69
PeARF17	PeARF19	0.654	1.3	71.04
PeARF17	PeARF20	0.794	2.63	67.91
PeARF17	PeARF21	0.867	3.3	81.31
PeARF17	PeARF22	0.792	1.22	76.93
PeARF17	PeARF23-X1	0.823	1.1	52.91
PeARF17	PeARF23-X2	0.818	1.6	77.37
PeARF17	PeARF24	0.906	2.26	72.64
PeARF17	PeARF25	0.874	2.78	94.43
PeARF17	PeARF26	0.69	1.72	31.08
PeARF17	PeARF27-X1	0.908	1.28	78.58
PeARF17	PeARF27-X2	0.707	3.16	58.04
PeARF17	PeARF28	0.885	1.97	75.74
PeARF17	PeARF29-X1	0.822	3.3	45.95
PeARF17	PeARF29-X2	0.861	1.75	50.69
PeARF17	PeARF30-X1	0.873	3.03	70.74
PeARF17	PeARF30-X2	0.689	2.4	33.18
PeARF17	PeARF31-X1	0.916	1.02	58.64
PeARF17	PeARF31-X2	0.921	3.03	65.13
PeARF17	PeARF32	0.861	2.4	80.92
PeARF17	PeARF33	0.937	2.02	73.1
PeARF17	PeARF34	0.833	2.55	94.87
PeARF18	PeARF19	0.703	3.37	66.64
PeARF18	PeARF20	0.706	3.35	42.23
PeARF18	PeARF21	0.685	2.91	67.21
PeARF18	PeARF22	0.792	1.26	94.16

PeARF18	PeARF23-X1	0.924	2.05	80.41
PeARF18	PeARF23-X2	0.936	1.6	44.12
PeARF18	PeARF24	0.886	2.63	59.52
PeARF18	PeARF25	0.895	3.36	77.73
PeARF18	PeARF26	0.817	1.85	62.18
PeARF18	PeARF27-X1	0.889	1.51	54.58
PeARF18	PeARF27-X2	0.888	1.99	87.11
PeARF18	PeARF28	0.849	3.2	82.03
PeARF18	PeARF29-X1	0.939	2.31	42.85
PeARF18	PeARF29-X2	0.81	3.47	36.67
PeARF18	PeARF30-X1	0.879	1.71	60.18
PeARF18	PeARF30-X2	0.927	3.39	69.91
PeARF18	PeARF31-X1	0.854	2.2	59.4
PeARF18	PeARF31-X2	0.89	3.18	52.14
PeARF18	PeARF32	0.798	3.04	72.87
PeARF18	PeARF33	0.679	2.67	87.58
PeARF18	PeARF34	0.776	2.5	46.24
PeARF19	PeARF20	0.679	2.08	51.55
PeARF19	PeARF21	0.861	1.41	73.03
PeARF19	PeARF22	0.872	2.5	57.79
PeARF19	PeARF23-X1	0.786	2.22	78.51
PeARF19	PeARF23-X2	0.917	1.98	54.36
PeARF19	PeARF24	0.72	1.17	46.86
PeARF19	PeARF25	0.938	2.83	64.73
PeARF19	PeARF26	0.778	1.63	58.85
PeARF19	PeARF27-X1	0.664	1.25	78.2

PeARF19	PeARF27-X2	0.827	3	93.64
PeARF19	PeARF28	0.922	3.07	63.15
PeARF19	PeARF29-X1	0.899	1.39	75.39
PeARF19	PeARF29-X2	0.669	1.7	44.94
PeARF19	PeARF30-X1	0.861	3.19	67.11
PeARF19	PeARF30-X2	0.823	3.42	45.59
PeARF19	PeARF31-X1	0.847	2.81	42.41
PeARF19	PeARF31-X2	0.692	1.36	87.23
PeARF19	PeARF32	0.788	1.15	56.94
PeARF19	PeARF33	0.879	2.27	47.36
PeARF19	PeARF34	0.838	1.51	34.36
PeARF20	PeARF21	0.765	2.72	33.87
PeARF20	PeARF22	0.9	1.78	92.89
PeARF20	PeARF23-X1	0.719	2.36	34.17
PeARF20	PeARF23-X2	0.929	3.1	77.62
PeARF20	PeARF24	0.737	1.69	60.69
PeARF20	PeARF25	0.704	1.55	49.74
PeARF20	PeARF26	0.935	1.43	73.38
PeARF20	PeARF27-X1	0.81	1.64	67.48
PeARF20	PeARF27-X2	0.892	2.97	92.96
PeARF20	PeARF28	0.779	1.07	69.67
PeARF20	PeARF29-X1	0.927	3.14	92.93
PeARF20	PeARF29-X2	0.703	2.98	58.21
PeARF20	PeARF30-X1	0.866	1	83.59
PeARF20	PeARF30-X2	0.822	1.5	90.02
PeARF20	PeARF31-X1	0.833	3.28	63.01

PeARF20	PeARF31-X2	0.718	1.25	38.05
PeARF20	PeARF32	0.881	2.91	93.5
PeARF20	PeARF33	0.761	1.93	45.09
PeARF20	PeARF34	0.675	3.27	35.52
PeARF21	PeARF22	0.92	3.44	56.88
PeARF21	PeARF23-X1	0.673	2.68	56.81
PeARF21	PeARF23-X2	0.883	2.54	70.47
PeARF21	PeARF24	0.823	1.14	73.87
PeARF21	PeARF25	0.864	3.32	45.8
PeARF21	PeARF26	0.886	2.24	74.32
PeARF21	PeARF27-X1	0.86	3.35	63.14
PeARF21	PeARF27-X2	0.747	2.35	84.38
PeARF21	PeARF28	0.792	2.3	92.54
PeARF21	PeARF29-X1	0.742	2.39	56.51
PeARF21	PeARF29-X2	0.859	2.62	73.97
PeARF21	PeARF30-X1	0.914	3.05	37.7
PeARF21	PeARF30-X2	0.753	2.24	36.64
PeARF21	PeARF31-X1	0.939	2.74	92.46
PeARF21	PeARF31-X2	0.739	3.46	49.06
PeARF21	PeARF32	0.813	1.18	67.33
PeARF21	PeARF33	0.757	2.26	90.49
PeARF21	PeARF34	0.894	1.79	86
PeARF22	PeARF23-X1	0.707	1.9	42.68
PeARF22	PeARF23-X2	0.749	2.73	78.4
PeARF22	PeARF24	0.937	2.02	89.53
PeARF22	PeARF25	0.931	2.82	71.78

PeARF22	PeARF26	0.745	2.36	64.74
PeARF22	PeARF27-X1	0.691	1.44	74.95
PeARF22	PeARF27-X2	0.659	1.49	66.88
PeARF22	PeARF28	0.702	2.11	86.87
PeARF22	PeARF29-X1	0.916	3.14	65.17
PeARF22	PeARF29-X2	0.792	1.34	36.18
PeARF22	PeARF30-X1	0.932	2.7	58.35
PeARF22	PeARF30-X2	0.669	2.7	86.96
PeARF22	PeARF31-X1	0.741	3.12	49.86
PeARF22	PeARF31-X2	0.674	2.21	41.46
PeARF22	PeARF32	0.78	2.83	31.79
PeARF22	PeARF33	0.863	3	93.91
PeARF22	PeARF34	0.663	3.45	57.02
PeARF23-X1	PeARF23-X2	0.788	2.22	65.45
PeARF23-X1	PeARF24	0.655	2.58	35.52
PeARF23-X1	PeARF25	0.653	1.33	61.33
PeARF23-X1	PeARF26	0.826	2.59	87.82
PeARF23-X1	PeARF27-X1	0.766	2.84	79.56
PeARF23-X1	PeARF27-X2	0.68	2.45	56.88
PeARF23-X1	PeARF28	0.672	1.79	82.19
PeARF23-X1	PeARF29-X1	0.784	1.8	37.62
PeARF23-X1	PeARF29-X2	0.781	3.34	58.35
PeARF23-X1	PeARF30-X1	0.75	1.8	50.56
PeARF23-X1	PeARF30-X2	0.708	2.12	71.2
PeARF23-X1	PeARF31-X1	0.947	1.78	52.01
PeARF23-X1	PeARF31-X2	0.787	1.68	81.58

PeARF23-X1	PeARF32	0.762	1.48	61.15
PeARF23-X1	PeARF33	0.716	1.95	73.89
PeARF23-X1	PeARF34	0.8	3.23	42.93
PeARF23-X2	PeARF24	0.762	2.39	63.16
PeARF23-X2	PeARF25	0.924	1.87	85.6
PeARF23-X2	PeARF26	0.909	1.14	83.15
PeARF23-X2	PeARF27-X1	0.845	3.25	72.01
PeARF23-X2	PeARF27-X2	0.845	2.03	47.91
PeARF23-X2	PeARF28	0.833	2.89	67.22
PeARF23-X2	PeARF29-X1	0.697	2.86	72.11
PeARF23-X2	PeARF29-X2	0.807	3.34	45.13
PeARF23-X2	PeARF30-X1	0.849	1.05	31.25
PeARF23-X2	PeARF30-X2	0.923	2.04	39.11
PeARF23-X2	PeARF31-X1	0.792	2.3	31.96
PeARF23-X2	PeARF31-X2	0.723	2.48	84.76
PeARF23-X2	PeARF32	0.892	1.14	86.13
PeARF23-X2	PeARF33	0.942	2.01	62.36
PeARF23-X2	PeARF34	0.946	2.89	94.34
PeARF24	PeARF25	0.697	2.67	62.03
PeARF24	PeARF26	0.816	3.28	79.67
PeARF24	PeARF27-X1	0.871	1.87	43.97
PeARF24	PeARF27-X2	0.86	1.74	48.99
PeARF24	PeARF28	0.84	1.32	91.12
PeARF24	PeARF29-X1	0.73	3.43	53.69
PeARF24	PeARF29-X2	0.928	1.98	89.75
PeARF24	PeARF30-X1	0.932	2.6	48.14

PeARF24	PeARF30-X2	0.77	2.78	63.99
PeARF24	PeARF31-X1	0.798	1.46	44.04
PeARF24	PeARF31-X2	0.939	1.68	33.2
PeARF24	PeARF32	0.774	2.48	53.04
PeARF24	PeARF33	0.734	2.91	92.63
PeARF24	PeARF34	0.856	1.05	59.43
PeARF25	PeARF26	0.943	2.27	77.92
PeARF25	PeARF27-X1	0.827	2.54	67.42
PeARF25	PeARF27-X2	0.946	1.14	40.72
PeARF25	PeARF28	0.709	1.79	79.21
PeARF25	PeARF29-X1	0.812	2.44	93.03
PeARF25	PeARF29-X2	0.686	1.41	72.47
PeARF25	PeARF30-X1	0.805	1.98	81.09
PeARF25	PeARF30-X2	0.781	1.05	82.12
PeARF25	PeARF31-X1	0.693	3.38	62.93
PeARF25	PeARF31-X2	0.677	2.93	53.84
PeARF25	PeARF32	0.725	1.23	73.42
PeARF25	PeARF33	0.868	2.02	71.49
PeARF25	PeARF34	0.882	3.14	72.9
PeARF26	PeARF27-X1	0.814	3.16	83.08
PeARF26	PeARF27-X2	0.667	2.25	81.95
PeARF26	PeARF28	0.779	1.8	39.82
PeARF26	PeARF29-X1	0.682	2.85	47.23
PeARF26	PeARF29-X2	0.839	3.14	80.36
PeARF26	PeARF30-X1	0.702	1.58	71.4
PeARF26	PeARF30-X2	0.936	2.5	61.5

PeARF26	PeARF31-X1	0.671	2.27	75.25
PeARF26	PeARF31-X2	0.817	1.22	87.13
PeARF26	PeARF32	0.812	1.39	33.36
PeARF26	PeARF33	0.937	1.46	60.48
PeARF26	PeARF34	0.656	2.47	48.56
PeARF27-X1	PeARF27-X2	0.934	2.22	31.22
PeARF27-X1	PeARF28	0.797	1.27	59.85
PeARF27-X1	PeARF29-X1	0.792	1.89	94.11
PeARF27-X1	PeARF29-X2	0.845	1.54	86.43
PeARF27-X1	PeARF30-X1	0.721	2.91	33.16
PeARF27-X1	PeARF30-X2	0.786	2.89	60.58
PeARF27-X1	PeARF31-X1	0.766	1.8	69.98
PeARF27-X1	PeARF31-X2	0.873	2	39.56
PeARF27-X1	PeARF32	0.737	1.91	77.34
PeARF27-X1	PeARF33	0.678	2	62.4
PeARF27-X1	PeARF34	0.864	1.91	48.31
PeARF27-X2	PeARF28	0.929	2.35	45.98
PeARF27-X2	PeARF29-X1	0.909	3.04	47.44
PeARF27-X2	PeARF29-X2	0.88	2.22	93.54
PeARF27-X2	PeARF30-X1	0.68	2.47	47.99
PeARF27-X2	PeARF30-X2	0.688	2.45	79.23
PeARF27-X2	PeARF31-X1	0.851	1.97	70.23
PeARF27-X2	PeARF31-X2	0.798	3.32	92.88
PeARF27-X2	PeARF32	0.686	2.85	74.67
PeARF27-X2	PeARF33	0.933	2.81	89.5
PeARF27-X2	PeARF34	0.895	1.76	81.75

PeARF28	PeARF29-X1	0.725	2.34	54.09
PeARF28	PeARF29-X2	0.724	3	94.89
PeARF28	PeARF30-X1	0.782	1.93	54.76
PeARF28	PeARF30-X2	0.895	1.11	43.49
PeARF28	PeARF31-X1	0.93	2.41	78.73
PeARF28	PeARF31-X2	0.727	2.67	85.44
PeARF28	PeARF32	0.702	2.68	65.48
PeARF28	PeARF33	0.811	3.04	61.94
PeARF28	PeARF34	0.786	2.68	89.68
PeARF29-X1	PeARF29-X2	0.736	3.1	34.9
PeARF29-X1	PeARF30-X1	0.695	3.23	35.88
PeARF29-X1	PeARF30-X2	0.851	2.82	71.31
PeARF29-X1	PeARF31-X1	0.715	3.25	46.73
PeARF29-X1	PeARF31-X2	0.732	2.83	63.39
PeARF29-X1	PeARF32	0.737	2.55	79.55
PeARF29-X1	PeARF33	0.881	1.8	45.97
PeARF29-X1	PeARF34	0.679	1.5	34.34
PeARF29-X2	PeARF30-X1	0.77	3.35	49.35
PeARF29-X2	PeARF30-X2	0.884	3	33.88
PeARF29-X2	PeARF31-X1	0.762	3.36	87.18
PeARF29-X2	PeARF31-X2	0.841	2.2	35.69
PeARF29-X2	PeARF32	0.758	1.04	82.35
PeARF29-X2	PeARF33	0.872	1.24	72.03
PeARF29-X2	PeARF34	0.9	1.42	42.78
PeARF30-X1	PeARF30-X2	0.923	1.27	38
PeARF30-X1	PeARF31-X1	0.817	2.02	85.85

PeARF30-X1	PeARF31-X2	0.893	1.62	38.11
PeARF30-X1	PeARF32	0.758	2.95	84.19
PeARF30-X1	PeARF33	0.847	2.83	62.17
PeARF30-X1	PeARF34	0.906	1.2	48.41
PeARF30-X2	PeARF31-X1	0.74	3.49	76.36
PeARF30-X2	PeARF31-X2	0.896	1.9	52.79
PeARF30-X2	PeARF32	0.822	3.31	64.84
PeARF30-X2	PeARF33	0.695	3	43.14
PeARF30-X2	PeARF34	0.683	2.09	85.08
PeARF31-X1	PeARF31-X2	0.739	2.48	49.46
PeARF31-X1	PeARF32	0.941	1.39	80.4
PeARF31-X1	PeARF33	0.814	2.1	60.7
PeARF31-X1	PeARF34	0.848	2.51	46.6
PeARF31-X2	PeARF32	0.924	1.05	83.11
PeARF31-X2	PeARF33	0.839	2.78	77.78
PeARF31-X2	PeARF34	0.82	2.88	74.34
PeARF32	PeARF33	0.899	1.4	58.16
PeARF32	PeARF34	0.802	1.75	82.02
PeARF33	PeARF34	0.878	1.89	67.28

Table S4. Prediction of subcellular localization of ARF family members in *P. euphratica*

**Protein ID**	**Main Predicted location**	**Score**	**Other locations (Score)**
PeARF1	nucleus	14	-
PeARF2 X1	nucleus	11	cytoplasm(1),vacuole(1), cytoskeleton(1)
PeARF2 X2	nucleus	11	cytoplasm(1), vacuole(1), cytoskeleton(1)
PeARF2 X3	nucleus	11	cytoplasm(1), vacuole(1), cytoskeleton(1)

PeARF2 X4	nucleus	11	cytoplasm(1), vacuole(1), cytoskeleton(1)
PeARF3 X1	nucleus	12	plasma membrane(1), cytoskeleton(1)
PeARF3 X2	nucleus	13	cytoskeleton(1)
PeARF4 X1	nucleus	10	chloroplast(3), cytoskeleton(1)
PeARF4 X2	nucleus	10	chloroplast(3), cytoskeleton(1)
PeARF5	nucleus	10	chloroplast(2), cytoplasm(2)
PeARF6	nucleus	14	-
PeARF7 X1	nucleus	14	-
PeARF7 X2	nucleus	14	-
PeARF8	nucleus	11	cytoplasm(2), cytoskeleton(1)
PeARF9	nucleus	11	cytoplasm(2), cytoskeleton(1)
PeARF10	nucleus	8	chloroplast(3), extracellular(1), endoplasmic reticulum(1),cytoskeleton(1)
PeARF11 X1	nucleus	11	cytoskeleton(2), cytoplasm(1)
PeARF11 X2	nucleus	11	cytoskeleton(2), cytoplasm(1)
PeARF12	nucleus	11	cytoskeleton(2), cytoplasm(1)
PeARF13 X1	nucleus	13	cytoskeleton(1)
PeARF13 X2	nucleus	13	cytoskeleton(1)
PeARF14 X1	nucleus	10	chloroplast(3), endoplasmic reticulum(1)
PeARF14 X2	nucleus	9	chloroplast(3), cytoplasm(1), endoplasmic reticulum(1)
PeARF15	nucleus	14	-
PeARF16 X1	nucleus	8	cytoplasm(4), cytoskeleton(2)
PeARF16 X2	nucleus	11	cytoplasm(1), vacuole(1), golgi apparatus(1)
PeARF16 X3	nucleus	10	cytoplasm(2), vacuole(1), cytoskeleton(1)
PeARF16 X4	nucleus	9	cytoplasm(2), cytoskeleton(2), chloroplast(1)
PeARF16 X5	nucleus	9	cytoplasm(2), cytoskeleton(2), chloroplast(1)

PeARF17	nucleus	13	vacuole(1)
PeARF18 X1	nucleus	11	cytoplasm(1), plasma membrane(1), vacuole(1)
PeARF18 X2	nucleus	11	cytoplasm(1), plasma membrane(1), vacuole(1)
PeARF18 X3	nucleus	11	cytoplasm(1), plasma membrane(1), vacuole(1)
PeARF19	nucleus	14	-
PeARF20 X1	nucleus	10	cytoplasm(2), cytoskeleton(2)
PeARF20 X2	nucleus	10	cytoplasm(2), cytoskeleton(2)
PeARF21	nucleus	13	cytoplasm(1)
PeARF22	nucleus	14	-
PeARF23 X1	nucleus	13	vacuole(1)
PeARF23 X2	nucleus	13	vacuole(1)
PeARF24	nucleus	13	cytoplasm(1)
PeARF25	nucleus	14	-
PeARF26	nucleus	14	-
PeARF27 X1	nucleus	11	cytoplasm(2), cytoskeleton(1)
PeARF27 X2	nucleus	11	cytoplasm(2), golgi apparatus(1)
PeARF27 X3	nucleus	11	cytoplasm(2), golgi apparatus(1)
PeARF28	nucleus	14	-
PeARF29 X1	nucleus	13	vacuole(1)
PeARF29 X2	nucleus	13	vacuole(1)
PeARF30 X1	nucleus	14	-
PeARF30 X2	nucleus	14	-
PeARF31 X1	nucleus	9	chloroplast(2), cytoskeleton(2), plasma membrane(1)

PeARF31 X2	nucleus	10	plasma membrane(1.5), golgi apparatus(1.5),chloroplast(1), cytoskeleton(1)
PeARF32	nucleus	13	chloroplast(1)
PeARF33	nucleus	12	cytoplasm(1), plasma membrane(1)
PeARF34	nucleus	11	cytoplasm(2), chloroplast(1)

Table S5. Frequency and proportion of hormone-, light-, and stress-responsive cis-acting elements in the promoter regions of PeARF genes

Gene	Hormone-responsive	Light-responsive	Stress-related	Other	Total
PeARF1	6(4.11%)	5(3.42%)	11(7.53%)	124(84.93%)	146
PeARF2	6(4.44%)	7(5.19%)	9(6.67%)	113(83.7%)	135
PeARF3	10(5.62%)	11(6.18%)	3(1.69%)	154(86.52%)	178
PeARF4	8(6.56%)	7(5.74%)	5(4.10%)	102(83.61%)	122
PeARF5	13(8.44%)	12(7.79%)	11(7.14%)	118(76.62%)	154
PeARF6	7(4.32%)	6(3.70%)	9(5.56%)	140(86.42%)	162
PeARF7	11(5.37%)	12(5.85%)	10(4.88%)	172(83.9%)	205
PeARF8	2(1.45%)	6(4.35%)	15(10.87%)	115(83.33%)	138
PeARF9	17(9.88%)	19(11.05%)	6(3.49%)	130(75.58%)	172
PeARF10	5(2.89%)	10(5.78%)	13(7.51%)	145(83.82%)	173
PeARF11	4(2.72%)	14(9.52%)	8(5.44%)	121(82.31%)	147
PeARF12	4(2.55%)	14(8.92%)	8(5.10%)	131(83.44%)	157
PeARF13	12(7.10%)	11(6.51%)	7(4.14%)	139(82.25%)	169
PeARF14	9(5.66%)	14(8.81%)	10(6.29%)	126(79.25%)	159
PeARF15	8(5.19%)	4(2.60%)	12(7.79%)	130(84.42%)	154
PeARF16	12(7.27%)	10(6.06%)	7(4.24%)	136(82.42%)	165
PeARF17	6(4.44%)	6(4.44%)	11(8.15%)	112(82.96%)	135

PeARF18	13(9.56%)	8(5.88%)	11(8.09%)	104(76.47%)	136
PeARF19	1(0.74%)	11(8.09%)	6(4.41%)	118(86.76%)	136
PeARF20	9(6.62%)	7(5.15%)	5(3.68%)	115(84.56%)	136
PeARF21	9(5.29%)	8(4.71%)	9(5.29%)	144(84.71%)	170
PeARF22	12(5.50%)	10(4.59%)	7(3.21%)	189(86.7%)	218
PeARF23	9(5.00%)	10(5.56%)	12(6.67%)	149(82.78%)	180
PeARF24	16(10.60%)	8(5.30%)	11(7.28%)	116(76.82%)	151
PeARF25	10(6.76%)	11(7.43%)	6(4.05%)	121(81.76%)	148
PeARF26	8(5.56%)	8(5.56%)	6(4.17%)	122(84.72%)	144
PeARF27	11(8.46%)	10(7.69%)	3(2.31%)	106(81.54%)	130
PeARF28	5(3.45%)	10(6.90%)	10(6.90%)	120(82.76%)	145
PeARF29	11(7.69%)	8(5.59%)	10(6.99%)	114(79.72%)	143
PeARF30	9(5.14%)	9(5.14%)	8(4.57%)	149(85.14%)	175
PeARF31	10(6.58%)	11(7.24%)	7(4.61%)	124(81.58%)	152
PeARF32	5(3.57%)	9(6.43%)	7(5.00%)	119(85.0%)	140
PeARF33	7(4.35%)	6(3.73%)	7(4.35%)	141(87.58%)	161
PeARF34	14(5.69%)	14(5.69%)	10(4.07%)	208(84.55%)	246
Total	299(5.56%)	326(6.06%)	290(5.39%)	4467(83.00%)	5382
